# Mitochondria-specific targeting of noncanonical *EGR1* ntmRNA-coordinated mitophagy receptor BNIP3 homodimerization disrupts mitochondrial metabolism and suppresses hepatocellular carcinoma growth *in vitro* and *in vivo*

**DOI:** 10.7150/thno.117745

**Published:** 2026-01-01

**Authors:** Yan Li, Lei Zhou, Mengmeng Liu, Hui Li, Ying Meng, Xue Wen, Yijing Zhao, Shanshan Liu, Zi Yan, Changhao Fu, Shan Zong, Wei Li, Andrew R. Hoffman, Jiuwei Cui, Ji-Fan Hu

**Affiliations:** 1 Cancer Center, First Hospital of Jilin University, Changchun, Jilin 130061, China.; 2 Stanford University Medical School, Stanford, CA 94305, USA.

**Keywords:** mitophagy, *EGR1* ntmRNA, BNIP3 dimerization, mitochondria, hypoxia, hepatocellular carcinoma

## Abstract

**Background:** Hypoxia-driven metabolic reprogramming is a hallmark of hepatocellular carcinoma (HCC) and depends critically on mitochondrial signaling. We sought to identify RNA-based molecular factors that orchestrate the hypoxia-mitochondria crosstalk and regulate metabolic adaptation in HCC cells.

**Methods:** An integrated mtRNA-seq and mitochondria-specific LwaCas13a-BN-MLS RNA targeting approach was employed to profile RNA molecules aberrantly enriched in HCC mitochondria. Mitophagy was assessed via mt-Keima assay, immunofluorescence, transmission electron microscopy, and Western blotting of key autophagic markers. RNA-protein interactions were examined using RNA immunoprecipitation (RIP), electrophoretic mobility shift assays (EMSA), and computational structural modeling. *In vitro* and* in vivo* tumorigenicity was evaluated using colony formation, transwell invasion, wound healing, and subcutaneous xenograft models in nude mice.

**Results:** Nuclear-encoded *EGR1* mRNA was aberrantly translocated to mitochondria, where it functions as a non-translating mRNA (ntmRNA) essential for mitophagy. Mitochondria-specific *EGR1* targeting disrupted mitochondrial homeostasis by accumulating damaged mitochondria, lowering ATP, increasing ROS, reducing membrane potential, diminishing spare respiratory capacity, and impairing hypoxia-induced mitophagy. Mechanistically, *EGR1* ntmRNA promoted mitophagy through the HIF-1α/BNIP3/NIX axis by recruiting BNIP3 to mitochondria and coordinating its homodimerization via a 3′-UTR MRE. A synthetic MRE oligonucleotide rescued BNIP3 dimerization after *EGR1* depletion. Finally, we demonstrated that *EGR1* loss suppressed malignant phenotypes *in vitro* and reduced xenograft tumor growth *in vivo*.

**Conclusions:** This study reveals a noncanonical role for *EGR1* mRNA as an epigenetic regulator of mitophagy in HCC, thus expanding the functional repertoire of mRNA molecules beyond protein coding. Targeting this noncanonical* EGR1* ntmRNA-BNIP3 homodimerization mechanism may suggest new therapeutic strategies for treating HCC.

## Introduction

The hypoxic tumor microenvironment plays a critical role in determining malignant aggressiveness, therapeutic resistance to conventional chemotherapeutic drugs, and poor survival in a variety of human malignancies 1-3, including hepatocellular carcinoma 4. To survive hypoxic stress, cancer cells initiate a comprehensive epigenetic re-writing of many transcriptional and translational programs, enabling cell survival, tumor metastasis and resistance to anti-cancer therapeutics 5. Hypoxic niches drive genetic instability and create a heterogeneous population of malignant cells, including cancer stem cells 6, 7, exhibiting pro-survival and pro-invasive aggressive characteristics mediated by a HIF-1-coordinated extracellular and intracellular regulatory crosstalk network 8-11.

Extracellularly, hypoxia within the tumor microenvironment modifies the activity of infiltrating cells towards immunosuppressive phenotypes through the interplay of tumor-associated macrophages (TAMs), cancer-associated fibroblasts (CAFs), and infiltrated dendritic cells and T-cells. This modification empowers cancer cells to survive and evade immune attack 12-14. The accumulation of acidic metabolites, such as the high levels of lactate from enhanced glycolytic flux, diminishes immune cell activity by altering the antigen-presenting capacity, T-cell activation, migration, and cytokine release. Hypoxia also favors the activation of an M2-like phenotype in tumor infiltrating macrophages, promoting an anti-inflammatory response and promoting tumor growth. From this point of view, targeting tumor infiltrating CAFs and TAMs has emerged as a potential therapeutic strategy to unleash the host immune system against the tumor 15, 16. This emerging concept has currently been tested in clinical trials using small molecules and antibody therapeutics 17, 18.

Intracellularly, hypoxic stress induces metabolic reprogramming through the HIF-1 coordinated gene network 19-21, leading to a shunting of glucose and fatty acid metabolites away from the mitochondria and an increasing glucose and amino acid uptake, glycolytic flux, and lactate production 22-25. As a result of this metabolic switch, cells exhibit malignant phenotypes involving glutamine metabolism, the tricarboxylic acid cycle, oxidative phosphorylation, as well as fatty acid synthesis and oxidation. In parallel, cells generate high levels of mitochondrial reactive oxygen species. As a critical cell stress sensor, mitochondria play a vital role in this metabolic reprogramming 26-28.

However, the molecular factors that signal hypoxic stress to the mitochondria of HCC cells remain unknown 29. We utilized mitochondria-specific LwaCas13a-BN-MLS RNA targeting in combination with mitochondrial RNA-seq (mtRNA-seq) to identify key RNA components in the mitochondria of HCC cells that orchestrate the hypoxia-related metabolic switch. Using this approach, we found that in addition to long noncoding RNAs, such as *MALAT1* 30, 31, the early growth response protein 1 (*EGR1*) was listed as a top aberrantly enriched mRNA within the mitochondria of HCC cells. These mitochondria-enriched mRNAs did not translate into EGR1 protein, and thus were named *EGR1* non-translating mRNA (ntmRNA). *EGR1* is encoded by the nuclear genome, and it plays an important role in mediating the response to hypoxic and ischemic stress. In this communication, we examine the role of* EGR1* ntmRNA molecules in mitochondria, focusing on the regulation of mitochondrial metabolic reprogramming. Our data provide the first evidence that, in addition to the canonical coding function of* EGR1* peptides to initiate the immediate early response to stress 32-34, the nuclear-encoded* EGR1* ntmRNAs aberrantly translocate to HCC mitochondria, where they function as a new class of epigenetic ntmRNA messengers and regulate mitophagy in HCC cells by coordinating the homodimerization of mitophagy receptor BNIP3, a critical event for mitophagy initiation. These studies suggest that mRNAs can act like noncoding RNAs in regulating cell metabolism. Targeting *EGR1* ntmRNA-BNIP3 homodimerization may provide new therapeutic strategies for treating HCC.

## Results

### Mitochondrial Cas13a-mBN-MLS RNA targeting and mtRNA-seq reveal the enriched EGR1 ntmRNA in the mitochondria of hepatoma cells

**Figure 1:**
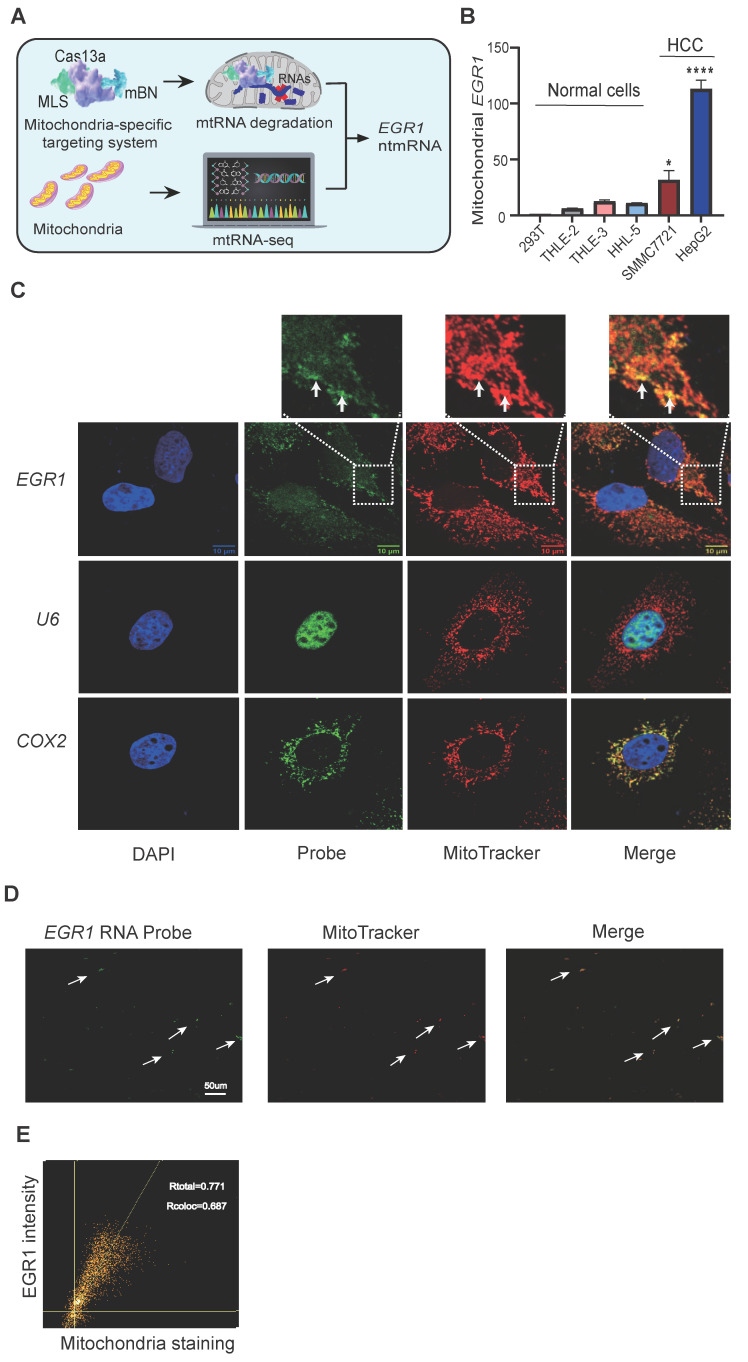
** The nuclear-encoded *EGR1* mRNA is enriched in mitochondria of hepatoma cells. A** Identification of the mitochondria-enriched *EGR1* ntmRNA using Cas13a-mBN-MLS mitochondria-specific targeting and mitochondrial RNA-seq. Cas13a: LwaCas-13a RNA-excision enzyme; gRNA: Cas13a guiding RNA; mBN: the mutant Barnase carrying D54A and H102A mutations. MLS: mitochondria-localization signal peptide derived from COX8A. The mitochondria-targeting system combines the advantages of RNA targeting specificity of Cas13a-gRNA, the RNase activity of mBN, and the mitochondria localization of MLS. The attachment of MLS facilitates the specific localization of the Cas13a-mBN in the mitochondria. The potent RNase activity of mBN efficiently degrades the RNA target in the mitochondria. This strategy identifies the mitochondria-enriched *EGR1* ntmRNA that functions as non-translating epigenetic messengers essential for mitophagy in HCC cells. **B** Differential mitochondria-enrichment of* EGR1* ntmRNA in hepatoma cells. HepG2 and SMMC7721: hepatoma cells; THLE-2, THLE-3 and HHL-5: normal hepatic cells; 293T: normal human embryonic kidney cells. The Ct values were normalized over that of *COX2* and then standardized by setting 293T as 1 for comparison. **C** RNA-FISH of* EGR1* in HepG2 cells. Cell nuclei were stained with DAPI (blue). *EGR1* mRNA was probed with Dig-labeled single-stranded DNA probes and was detected with FITC-coupled anti-dig antibody (green). Mitochondria were labeled with MitoTracker (red). The overlapping portion of *EGR1* mRNA and MitoTracker dye appears in yellow (enlarged windows, white arrows). The nuclear U6 was used as a negative control and the mitochondrial COX2 was used as a positive control. Scale bar: 10μm. **D** Co-localization of* EGR1* mRNA and Mito-Tracker in isolated mitochondria smear. Mitochondria were isolated from HepG2 cells and were smeared on slides. RNA-FISH was performed to detect *EGR1* mRNA (green). Mitochondria were tracked by MitoTracker staining (red). The co-localization portion of *EGR1* mRNA and mitochondria is displayed in yellow (white arrows). Scale bar: 50umμm. **E** Scatter plot of* EGR1* and Mito-Tracker. Pearson's correlation coefficient (PCC) was calculated for the entire image (Rtotal) and the pixels above thresholds (Rcoloc). 1: perfect correlation; -1: completely excluded; 0: random relationship. Data represents the mean ± SEM of three independent experiments. Significant differences were determined by an unpaired two-tailed *t*-test. * *p* < 0.05, **** *p* < 0.0001.

A mitochondria-specific RNA targeting and sequencing approach identified RNA components critical for hypoxia-related metabolic shifts in HCC mitochondria (**Figure 1A**). Mitochondrial RNA-seq (mtRNA-seq) first revealed enriched mitochondrial RNAs. Functional roles of these RNAs were explored using a novel mitochondria-targeted Cas13a-mBN-MLS system 35, 36, comprising RNA-targeting Cas13a fused to a mutant RNase Barnase (mBN) and mitochondria localization signal (MLS) from COX8A 37-39 (**Figure S1A-B**).

MtRNA-seq identified nuclear-encoded *EGR1* mRNA, an immediate-early gene, as notably enriched in hepatoma mitochondria (**Figure S1C**). Mitochondria-specific depletion of *EGR1* via Cas13a-gRNA-mBN-MLS impaired mitochondrial function. *EGR1* mRNAs were not translated into peptides in mitochondria but functioned as non-translating mRNAs (ntmRNA) essential for mitophagy in HCC cells. Therefore, we focused on its metabolic role in HCC cells.

We confirmed aberrant mitochondrial localization of *EGR1* ntmRNA. Quantitative PCR (**Figure S2A**) and RT-PCR (**Figure S2B**) validated enrichment in isolated hepatoma mitochondria, using mitochondria-encoded COX2 as a positive and nuclear U6 as a negative control. *EGR1* ntmRNA was abundant in mitochondria from HepG2 and SMMC7721 cells but negligible in normal hepatic (THLE-2, THLE-3, HHL-5) and HEK293T cell mitochondria (**Figure 1B**).

RNA fluorescence *in situ* hybridization (RNA-FISH) showed mitochondrial colocalization of *EGR1* ntmRNA (green) and MitoTracker (red) in HepG2 cells (**Figure 1C, arrows**), while U6 RNA was nuclear-localized. Similar results were obtained in SMMC7721 cells (**Figure S2C-D**). In addition, the abundance of total cellular *EGR1* mRNA was also high in HCC cell lines as compared with normal hepatic cells (**Figure S2E**).

RNA-FISH on RNase A-treated isolated mitochondria further confirmed mitochondrial *EGR1* ntmRNA presence (**Figure 1D**). Quantification using ImageJ demonstrated clear colocalization of *EGR1* ntmRNA with mitochondria (**Figure 1E**).

Together, these findings demonstrate mitochondrial compartment-specific translocation of *EGR1* ntmRNA distinct from the cytoplasmic mRNA pool.

### Mitochondrial EGR1 ntmRNA does not act as a messenger RNA to encode EGR1 peptides

LncRNAs shuttle between nuclei and mitochondria as epigenetic messengers coordinating cell metabolism 40-43. Nuclear-encoded mitochondrial lncRNAs (e.g., RNRP, MALAT1) function as anterograde signals, while mitochondrial-encoded nuclear lncRNAs (e.g., lncCytB) act as retrograde signals. *EGR1* mRNA encodes a transcription factor mediating immediate-early stress responses; however, whether mitochondrial *EGR1* mRNA acts as an anterograde signal or encodes peptides remains unclear (**Figure S3A**).

Thus, we first examined if *EGR1* mRNAs, when shuttled to the mitochondria, could function as messenger RNA to encode an EGR1 peptide using the mitochondrial translation machinery. As an immediate-early response gene,* EGR1* is activated when the cell is exposed to hypoxic stress. We first determined whether mitochondrial *EGR1* mRNA translates into EGR1 protein. HepG2 cells were exposed to hypoxia, and mitochondrial fractions were isolated. Western blot analysis showed increased EGR1 protein in whole-cell extracts following hypoxia (**Figure S3B, lanes 5-6**), but no EGR1 protein was detected in mitochondrial fractions under normoxic or hypoxic conditions (**Figure S3B, lanes 1-3**). Thus, mitochondrial *EGR1* mRNA does not serve as a canonical messenger RNA.

We confirmed these findings by immunofluorescence staining for EGR1 protein, observing nuclear but no mitochondrial localization under both normoxic and hypoxic conditions (**Figure S3C**). Collectively, these results exclude mitochondrial translation of *EGR1* mRNA. Instead, *EGR1* exhibits dual roles under hypoxia: canonical nuclear-ribosomal translation into peptides mediating stress responses, and non-canonical mitochondrial ntmRNA functions contributing to nucleus-mitochondria crosstalk (**Figure S3A**).

### EGR1 ntmRNA maintains mitochondrial function in HCC cells

**Figure 2:**
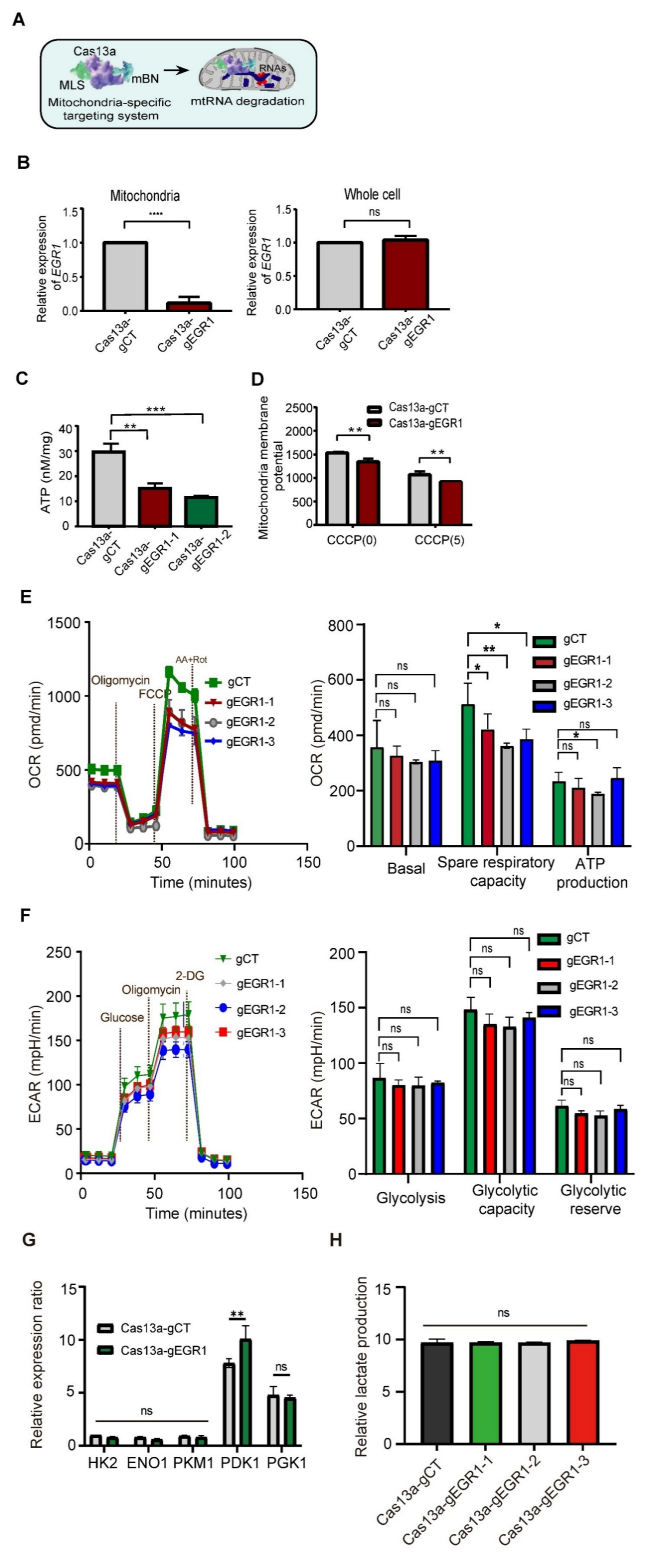
** Mitochondria-specific Cas13a-mBN-MLS targeting *EGR1* ntmRNA disrupts mitochondrial functions in HCC cells. A** Schematic diagram of the Cas13a-mBN-MLS targeting system. Cas13a: LwaCas-13a RNA-excision enzyme; gRNA: Cas13a guiding RNA; mBN: the mutant Barnase carrying D54A and H102A mutations. MLS: mitochondria-localization signal peptide derived from COX8A. The mitochondria-targeting system combines the advantages of RNA targeting specificity of Cas13a-gRNA, the RNase activity of mBN, and the mitochondria localization of MLS. The attachment of MLS facilitates the specific localization of the Cas13a-mBN in the mitochondria. **B** Quantitative PCR of *EGR1* mRNAs in the mitochondria using the LwaCas13a-mtBN-MLS system. Cas13a-gCT: random Cas13a gRNA; Cas13a-gEGR1: *EGR1*-specific Cas13a gRNA. Note the specific knockdown of *EGR1* in the mitochondria of HepG2 cells (left panel), while the level of *EGR1* mRNA in the whole cell was not significantly affected (right panel). **C** Reduction of ATP production in Cas13a-mBN-MLS-treated HepG2 cells. Two Cas13a gRNAs, Cas13a-gEGR1-1, Cas13a-gEGR1-2, were used to target mitochondria *EGR1* mRNAs. **D** Mitochondrial membrane potential in Cas13a-mBN-MLS-treated HepG2 cells. With or without the CCCP treatment, mitochondria were isolated from HCC cells for the measurement of mitochondrial membrane potential using fluorescent microplate reader. **E** The oxygen consumption rate (OCR) of *EGR1*-knockdown (Cas13a-gEGR1#1-3) and control (Cas13a-gCT) HepG2 cells were measured by the Seahorse. Specific knockdown of *EGR1* in the mitochondria of HepG2 cells reduces the OCR. Basal respiration, spare respiratory capacity, and ATP production were measured by Seahorse. **F** The extracellular cellular acidification rate (ECAR) of HepG2 cells were measured by the Seahorse. No difference in ECAR was observed between Cas13a-gCT group and Cas13a-gEGR1#1-3 group. Glycolysis, glycolytic capacity and glycolytic reserve were also measured by Seahorse. **G** The key enzyme of mitochondrial respiration HK2, ENO1, PKM1, PGK1 and PDK1 were measured by qPCR between Cas13a-gCT group and Cas13a-gEGR1 group. **H** Extracellular lactate assessment was performed in Cas13a-gCT group and mitochondrial *EGR1* targeting group (Cas13a-gEGR1-1, Cas13a-gEGR1-2, Cas13a-gEGR1-3). All data are presented as the mean ± S.D. from three independent experiments. Significant differences were determined by an unpaired two-tailed t-test (B-E) or two-way ANOVA with Tukey's multiple comparison test (G and H). * *p* < 0.05, ** *p* < 0.01, *** *p* < 0.001, **** *p* < 0.0001.

We next explored the non-canonical role of mitochondrial *EGR1* ntmRNA in HCC cells using Cas13a-mBN-MLS targeting (**Figure 2A**). This approach specifically depleted mitochondrial *EGR1* ntmRNA without significantly affecting cytoplasmic *EGR1* levels (**Figure 2B**). Targeted mitochondrial *EGR1* depletion significantly reduced ATP production (**Figure 2C**) and mitochondrial membrane potential in HepG2 cells, both under basal and CCCP-induced oxidative phosphorylation uncoupling conditions (**Figure 2D**).

Seahorse XF Cell Mito Stress assays showed that mitochondrial *EGR1* depletion reduced the spare respiratory capacity (**Figure 2E**) without significantly affecting glycolysis, as indicated by unchanged extracellular acidification rates (ECAR) (**Figure 2F**). Pyruvate dehydrogenase kinase 1 (PDK1), a key regulator linking glycolysis and mitochondrial oxidative phosphorylation, was upregulated upon mitochondrial *EGR1* targeting, whereas other glycolysis-related enzymes remain unaffected (**Figure 2G**). Despite increased PDK1 expression, extracellular lactate levels did not significantly change (**Figure 2H**). These data suggest that mitochondrial EGR1 depletion may trigger compensatory metabolic adaptations-such as pyruvate flux redistribution or post-translational regulation of glycolytic enzymes to maintain energetic homeostasis without enhancing overall glycolytic output.

We validated these results using lentiviral shRNAs (sh*EGR1*-1 and sh*EGR1*-2) to knock down *EGR1* expression (**Figure S4A**). Effective *EGR1* knockdown was confirmed in HepG2 (**Figure S4B**) and SMMC7721 (**Figure S5A**) cells, leading to similar reductions in ATP levels and mitochondrial membrane potential (**Figure S4C-D, Figure S5B-C**). These data collectively highlight mitochondrial *EGR1* ntmRNA as critical for maintaining mitochondrial function in HCC cells.

### Mitochondria-specific targeting of EGR1 ntmRNA induces the accumulation of reactive oxygen species

**Figure 3:**
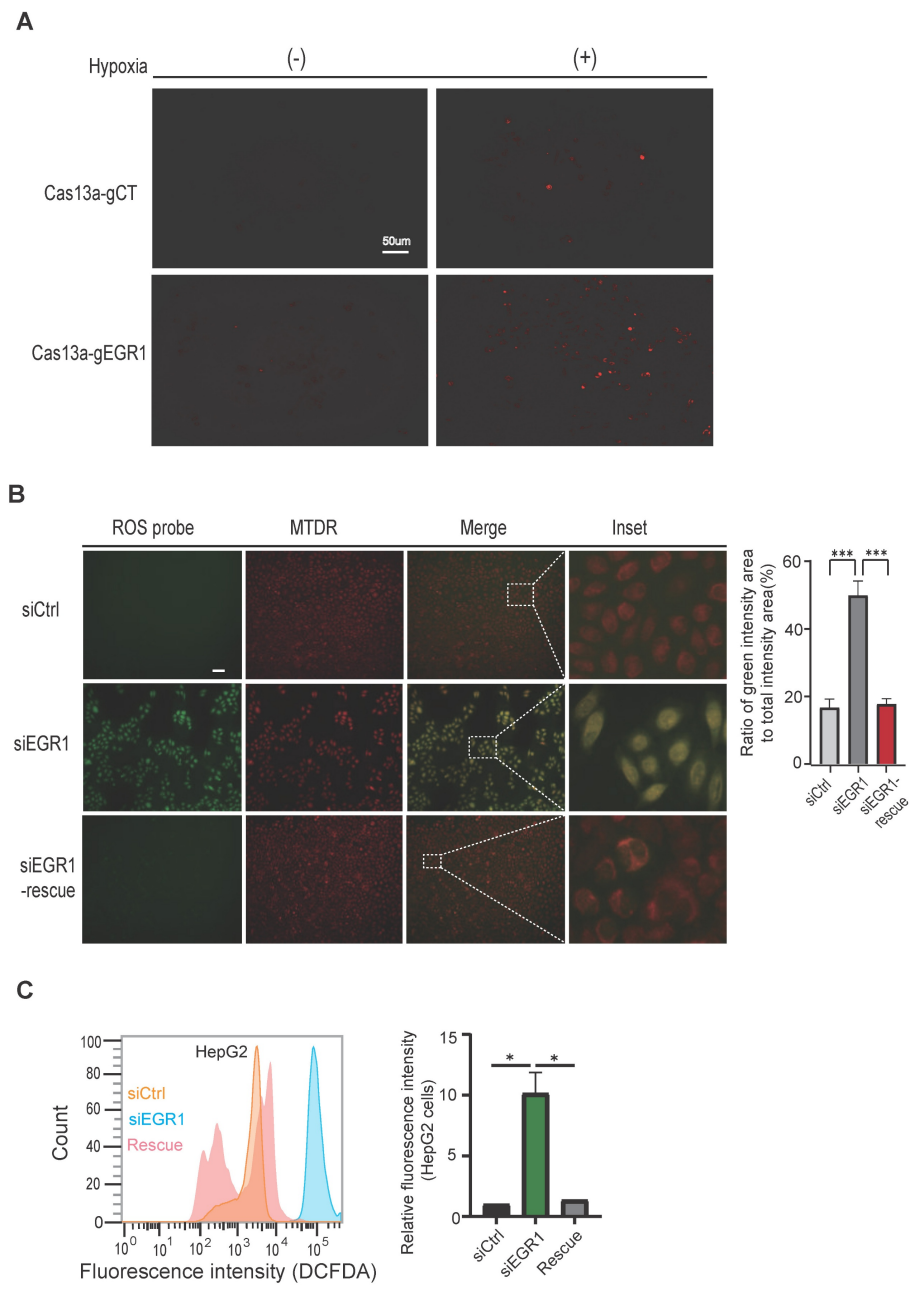
** Accumulation of reactive oxygen species in *EGR1* knockdown HepG2 cells. A** Reactive oxygen species (ROS) production in mitochondria was stained by MitoSOX™ (Molecular Probes). Cas13a-mBN-MLS-treated HepG2 cells and random Cas13a gRNA HepG2 cells were incubated under normoxic and hypoxic conditions for 24h and were then used for staining of ROS (red). **B** The rescue assay. SiCtrl control cells, siEGR1-treated cells and *EGR1*-rescue cells were cultured under hypoxic conditions for 12h and then used for ROS fluorescence staining using the ROS probe DCFDA/H2DCFDA (green), and MitoTracker Deep Red (MTDR). The yellow color indicates ROS within the mitochondria. Quantitative changes in the ROS green fluorescence intensity were analyzed by Image J. Scale bar: 20um. **C** Cellular ROS in HepG2 cells. ROS was examined using DCFDA probe followed by flow cytometry in the siCtrl (yellow), siEGR1 (blue) and siEGR1-rescue (pink) Groups.

Mitochondrial dysfunction often leads to increased reactive oxygen species (ROS) generation. To investigate whether mitochondrial EGR1 ntmRNA influences ROS production, we exposed HepG2 cells to hypoxia and used mitochondria-specific Cas13a-mBN-MLS targeting. Targeting mitochondrial EGR1 significantly elevated mitochondrial ROS levels (**Figure 3A, right bottom panel**).

We confirmed this finding using siRNA-mediated EGR1 knockdown in live HepG2 cells. Mitochondria were labeled with Mito-Tracker Deep Red (MTDR, red) and ROS were stained with DCFH-DA (green). The merged images (yellow) indicated mitochondrial ROS accumulation, which was significantly increased in EGR1-knockdown cells (**Figure 3B, middle panel**). Partial rescue of ROS accumulation was achieved by transfecting an EGR1 expression plasmid (**Figure 3B, bottom panel**). Similar results were obtained in SMMC7721 hepatoma cells (**Figure S5D, bottom panel**).

Flow cytometry with the ROS-sensitive probe DCFDA further confirmed elevated ROS levels in EGR1-knockdown HepG2 and SMMC7721 cells, which returned to near-control levels upon EGR1 rescue (**Figure 3C, Figure S5E**). These findings demonstrate that mitochondrial EGR1 ntmRNA plays an essential role in maintaining mitochondrial function and controlling ROS production under hypoxic stress in HCC cells.

### EGR1 ntmRNA is essential for hypoxia-mediated mitophagy

We next investigated the mechanism by which mitochondrial *EGR1* ntmRNA regulates mitochondrial function. Mitophagy is critical for removing dysfunctional mitochondria, maintaining mitochondrial integrity, and supporting metabolic reprogramming in cancer cells. Given that targeting mitochondrial *EGR1* impaired mitochondrial function and increased ROS production, we examined its impact on mitophagy in HCC.

First, we induced mitophagy by exposing HepG2 cells to hypoxia and assessed mitochondrial mass via immunofluorescence staining of TOM20, a mitochondrial import receptor subunit. Hypoxia reduced mitochondrial mass in control cells (Cas13a-gCT), whereas mitochondria-specific targeting of *EGR1* significantly inhibited this reduction (**Figure S6A, Figure S6C**). Western blot analysis confirmed these findings, showing reduced COX4 protein levels in hypoxia-treated control cells, which was blocked by mitochondria-specific *EGR1* depletion (**Figure S6B, Figure S6D**). Similarly, lentiviral shRNA-mediated *EGR1* knockdown also reduced hypoxia-induced TOM20 and COX4 degradation (**Figure S7A-D**), which was restored upon *EGR1* re-expression (**Figure S7C-D**). Thus, *EGR1* ntmRNA is essential for hypoxia-induced mitophagy.

**Figure 4:**
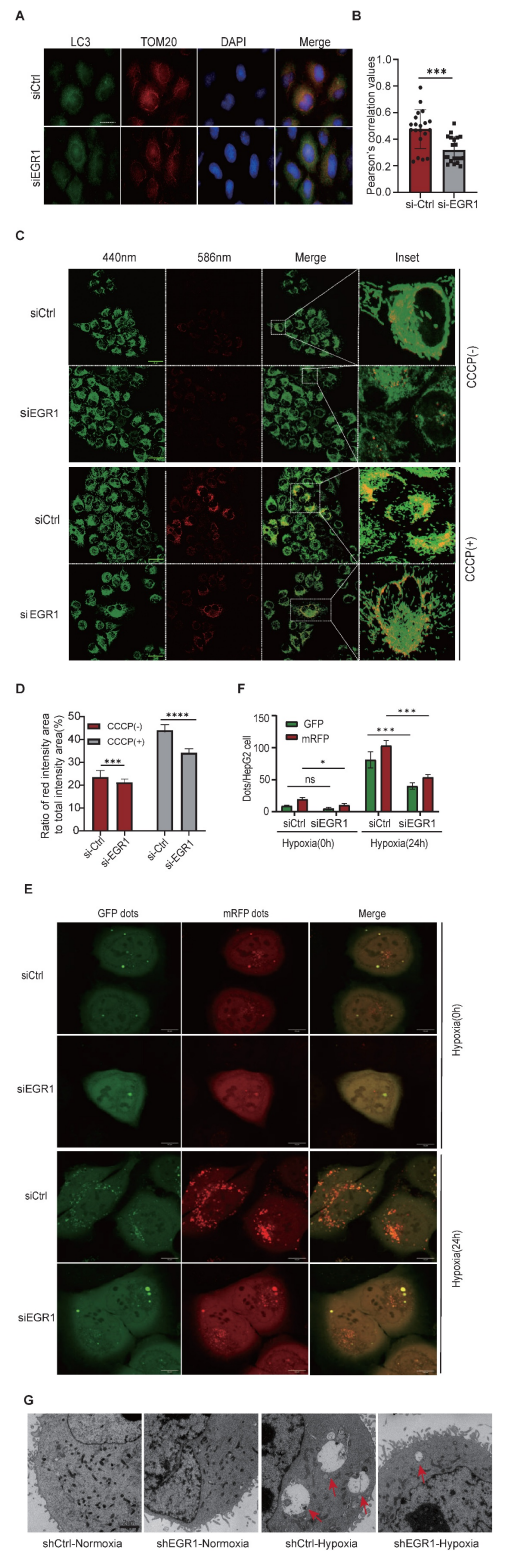
***EGR1* is essential for mitophagy. A** Colocalization of TOM20 and LC3 by immunofluorescent staining in siCtrl and siEGR1-treated HepG2 cells. Nuclei were stained with DPAI (blue), LC3 was immunostained in green, and TOM20 was immunostained in red. The overlapping portion of TOM20 and LC3 dye appears in yellow. Scale bar: 10μm. **B** Pearson's coefficient is shown as the quantification of LC3 puncta colocalized with TOM20 per cell in (A) (20 fields counted per group). **C** Mitophagy activity in HepG2 cells by the mt-Keima assay. Green dots represent the initial state of mitochondria, and red dots indicate mitochondria in lysosomes. A relatively increased ratio of red intensity area to total intensity area (red and green) suggests enhanced mitophagy. Scale bar: 50 μm. **D** Ratio of red intensity area to total intensity area (red and green) of mt-Keima from figure C (n = 4). **E** Changes in autophagic flux were observed in siCtrl and siEGR1 HepG2 cells under normoxic conditions and after 24-hour hypoxia treatment using confocal microscopy. The GFP signal (green) is quenched in the lysosomal environment, while the RFP signal (red) remains stable under acidic conditions. Therefore, autophagosomes (GFP⁺RFP⁺LC3 puncta) appear yellow (merged from green and red signals), and autolysosomes (GFP⁻RFP⁺LC3 puncta) appear red. By detecting and analyzing these distinct fluorescent signals, autophagic flux can be monitored. Scale bar: 10μm. **F** The manual counting method was used to quantify the number of red puncta and green puncta per HepG2 cell(n=3). **G** Transmission electron microscope (TEM) assay. HepG2 cells were exposed to hypoxia for 24 hours and were collected for the detection of autophagy-lysosomes under TEM. The hypoxia exposure induced the formation of autophagy-lysosomes engulfed with damaged mitochondria in shCtrl cells. Note the small and few autophagy-lysosomes in the sh*EGR1*-treated group as compared with the shCtrl group. Data represents the mean ± SEM of three independent experiments. Significant differences were determined by an unpaired two-tailed t-test (B,D) or two-way ANOVA with Tukey's multiple comparison test (F). *p < 0.05, **p < 0.01, *** *p* < 0.001, **** p < 0.0001.

Second, immunofluorescence staining revealed that colocalization of the autophagosome marker LC3 (green) with mitochondrial TOM20 (red) decreased significantly upon *EGR1* knockdown in HepG2 cells (**Figure 4A-B**), suggesting impaired mitophagy.

Third, using mt-Keima assays, we measured mitophagic flux following CCCP induction. CCCP treatment elevated mitophagy in control cells (siCtrl), as shown by increased red fluorescence. This enhanced mitophagic flux was significantly diminished in *EGR1* knockdown cells (**Figure 4C-D**). Additionally, HepG2 cells expressing mRFP-GFP-LC3 exhibited increased autolysosome formation (red puncta) under hypoxia, whereas *EGR1* knockdown markedly suppressed this process (**Figure 4E-F**), indicating reduced autophagic flux.

Finally, transmission electron microscopy (TEM) confirmed fewer autophagy-lysosome structures encapsulating damaged mitochondria in hypoxia-exposed *EGR1*-knockdown cells compared to control cells (**Figure 4G**). Notably, *EGR1* ntmRNA had minimal effects under normoxia, consistent with low basal mitophagy levels in hepatoma cells.

Together, these findings demonstrate that mitochondrial *EGR1* ntmRNA is critical for regulating hypoxia-induced mitophagy in hepatoma cells.

### EGR1 is essential for autophagosome formation during mitophagy

**Figure 5:**
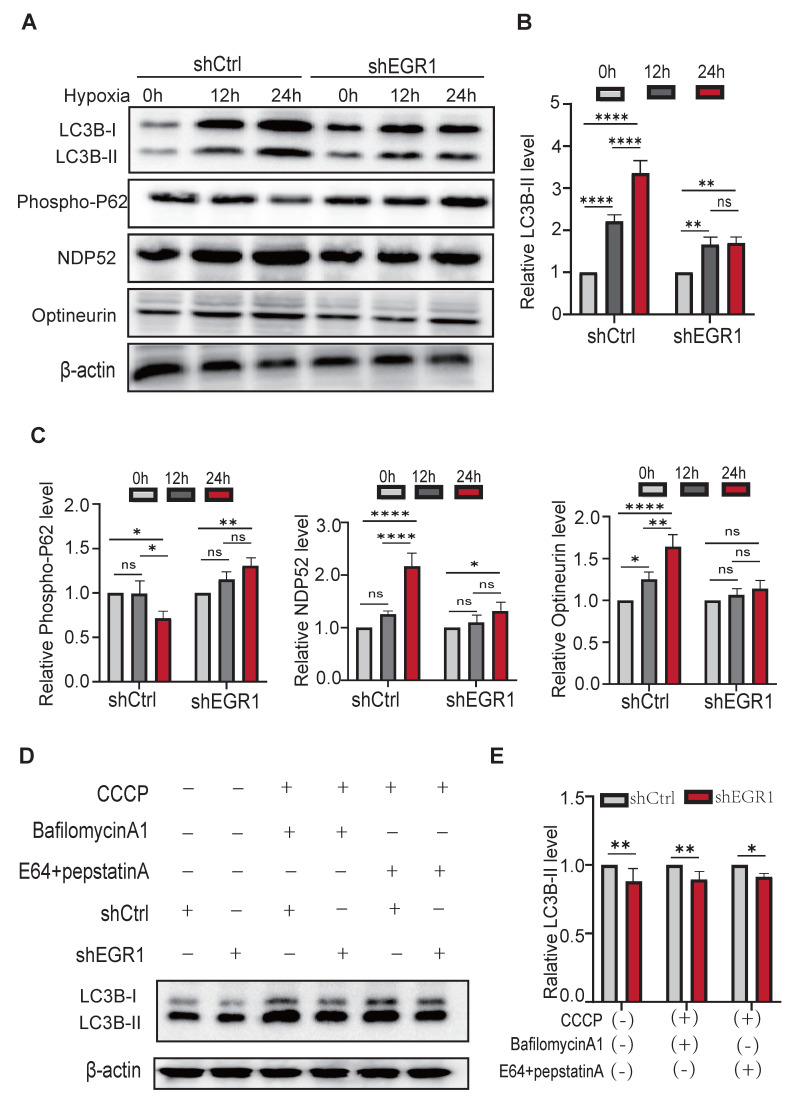
*** EGR1* is essential for autophagosome formation during mitophagy. A** Western blot of autophagosome formation proteins after 12h and 24h hypoxia. β-Actin was used as the control. **B** Quantitation of LC3B-II, normalized by the β-Actin level (*n* = 3 per group). **C** Quantitation of P62, NDP52, and Optineurin, normalized by β-Actin level (*n* = 3 per group). **D** Western blot of LC3B-II in the absence or presence of CCCP, BafilomycinA1, and E64+pepstatinA. **E** Quantitation of LC3B-II from figure D, normalized over the β-Actin level (*n* = 3 per group).

Under mitochondrial stress, autophagosomes selectively recruit cargo receptors, such as SQSTM1/p62, Optineurin, and NDP52, to target ubiquitinated mitochondrial proteins via LC3 interaction. To clarify the role of *EGR1* in autophagosome formation, we assessed LC3B-I/II, p62, Optineurin, and NDP52 protein levels following hypoxia. *EGR1* knockdown prevented hypoxia-induced p62 degradation and blocked accumulation of lipidated LC3B-II, Optineurin, and NDP52 (**Figure 5A; quantified in Figure 5B-C**), indicating impaired autophagic flux.

We further validated these results by inhibiting lysosomal degradation with bafilomycin A1 and pepstatin A + E64d (aloxistatin) (**Figure 5D**). Autophagic flux, assessed by LC3B-II accumulation, was significantly lower in *EGR1*-knockdown cells compared to controls, regardless of lysosomal inhibition (**Figure 5E**). Thus, reduced mitophagy in *EGR1*-deficient cells results primarily from impaired autophagosome formation rather than altered autophagolysosomal degradation.

### EGR1 regulates mitophagy through the HIF-1α/BNIP3/NIX pathway

**Figure 6:**
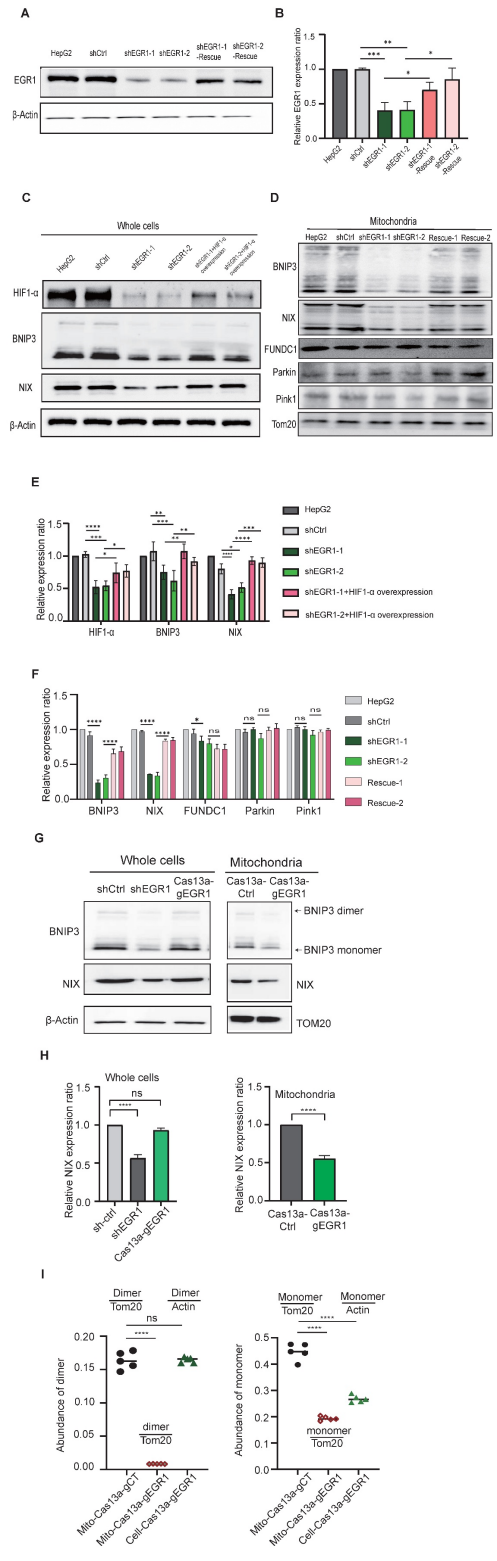
*** EGR1* knockdown inhibits mitophagy through HIF-1α/BNIP3/BNIP3L pathway. A** Western blot analysis was conducted to validate EGR1 protein knockdown efficiency and subsequent rescue. **B** Quantitation of Western blot results from A, normalized over the β-Actin level (*n* = 3 per group). **C** Western blot of HIF-1α protein and mitophagy pathway proteins in total cells. After 24h of hypoxic exposure, HepG2 cells, shCtrl cells, and *EGR1* knockdown combined with HIF-1α overexpression cells were collected for the measurement of mitophagy pathway proteins. β-Actin was used as the control. **D** Western blot of mitophagy pathway proteins from the isolated mitochondria. Tom20 was used as the control. **E** Quantitation of Western blot results from Figure C, normalized over the β-Actin level (*n* = 3 per group). **F** Quantitation of Western blot results from Figure D, normalized over the TOM20 level (*n* = 3 per group). **G** Western blot of BNIP3/NIX in total cells and isolated mitochondria. **H** Quantitation of Western blot results from Figure G, normalized over the β-Actin or TOM20 level (*n* = 3 per group). **I** Quantitation of BNIP3 dimer and monomer in Cas13a-mtBN-MLS targeted HepG2 cells, normalized over the β-Actin or TOM20 level (*n* = 5 per group). Data represents the mean ± SEM of three independent experiments. Significant differences were determined by two-way ANOVA with Tukey's multiple comparison test (E and F) or an unpaired two-tailed *t*-test (H) * *p* < 0.05, ** *p* < 0.01, *** *p* < 0.001, **** *p* < 0.0001.

Under hypoxia, cells activate hypoxia-inducible factor 1α (HIF-1α), triggering mitophagy via BNIP3/NIX signaling. We assessed the role of *EGR1* in this pathway by analyzing HIF-1α and downstream components in *EGR1*-knockdown HepG2 cells. Western blot confirmed effective *EGR1* knockdown (**Figure 6A-B**), reducing HIF-1α, BNIP3, and NIX protein levels, which were restored upon *EGR1* re-expression (**Figure 6C, Figure 6E**). Similarly, mitochondrial BNIP3/NIX proteins decreased significantly after *EGR1* knockdown but recovered upon *EGR1* rescue (**Figure 6D, Figure 6F**). Non-hypoxia-induced mitophagy regulators PINK1 and Parkin were unaffected (**Figure 6D, Figure 6F**).

Using Cas13a-mBN-MLS for mitochondria-specific targeting of *EGR1* ntmRNA, we found mitochondrial BNIP3/NIX expression was reduced without affecting cytoplasmic levels (**Figure 6G-H**). Additionally, mitochondrial *EGR1* depletion inhibited BNIP3 dimerization (**Figure 6G, Figure 6I**), a critical step for mitophagy initiation. It is known that BNIP3 dimerization within the ER membrane is essential for lysosomal trafficking in mitophagy initiation and progression 44-46. Thus, mitochondrial *EGR1* ntmRNA promotes mitophagy primarily by regulating BNIP3 dimerization through the HIF-1α/BNIP3/NIX pathway.

### EGR1 coordinates hypoxia-induced mitochondrial fission and fusion

Mitochondrial quality is regulated by balanced fission and fusion. During fission, phosphorylation of DRP1 at Ser616 and recruitment by mitochondrial fission factor (MFF) induce mitochondrial division, facilitating mitophagy. Conversely, phosphorylation at Ser637 inhibits DRP1-mediated fission, promoting mitochondrial fusion through MFN1/MFN2 and OPA1 47-49. Western blot analyses showed hypoxia-induced DRP1 (Ser616) and MFF upregulation were reduced by *EGR1* knockdown, while fusion markers DRP1 (Ser637), MFN1/MFN2, and OPA1 increased significantly (**Figure S8A,** quantified in **Figure S8B**). Thus, *EGR1* promotes mitophagy by enhancing mitochondrial fission and inhibiting fusion under hypoxia.

### EGR1 interacts with mitochondrial BNIP3 and NIX under hypoxic stimulation

**Figure 7:**
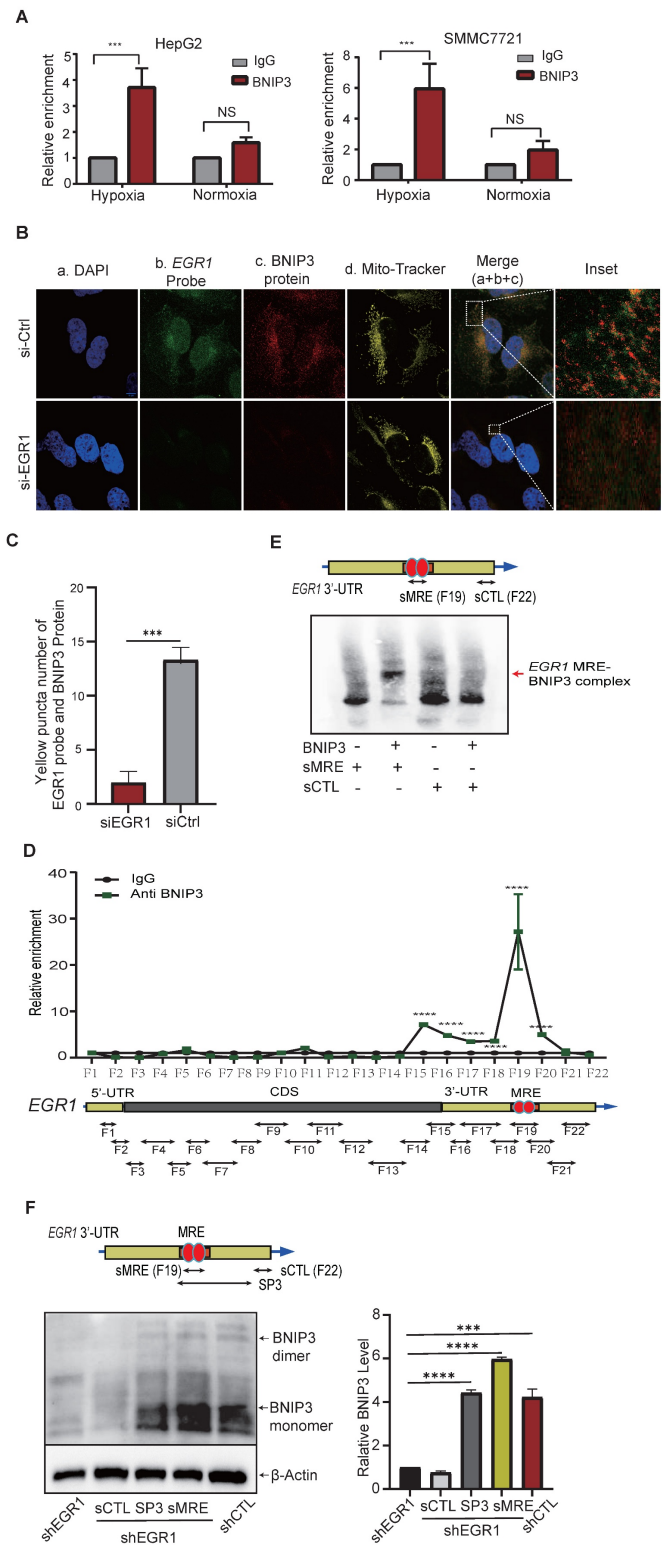
***EGR1* ntmRNA interacts with the mitophagy receptor BNIP3 in mitochondria. A*** EGR1* ntmRNA-BNIP3 interaction by the RIP assay. After exposure to hypoxia for 0h and 24h, HepG2 and SMMC7721 cells were collected, and the RIP assay was used to detect the interaction of *EGR1* mRNA with the mitophagy receptor BNIP3. *** p<0.001 as compared with the IgG control group. **B** Colocalization of *EGR1* mRNA with mitochondrial BNIP3 in HepG2 cells by RNA-FISH and immunofluorescent staining. The nuclei were stained with DAPI (blue) and mitochondria were labeled with MitoTracker (yellow). *EGR1* mRNA was probed with Dig-labeled single-stranded DNA probes and was detected with FITC-coupled anti-dig antibody (green). Anti-BNIP3 antibodies were used to label BNIP3 proteins (Red). The overlapping portion of *EGR1* mRNA with BNIP3 and NIX proteins appeared in yellow (enlarged windows). Scale bar: 10µm. **C** Quantitation of *EGR1* ntmRNA-BNIP3 interaction yellow puncta numbers in HepG2 cells (fluorescent scanning of Figure B) (n = 4). **D** Identification of the BNIP3 binding element by RIP mapping. The BNIP3 interacting* EGR1* RNA fragments were mapped by q-PCR using overlapping primers (bottom panel). For comparison, the value of the IgG control was set as 1. The F19 fragment contains a mitophagy receptor-binding element (MRE) to recruit BNIP3. **E** Synthetic *EGR1* MRE (sMRE) bound to BNIP3 *in vitro* by Electrophoretic Mobility Shift Assay (EMSA). Red arrow: the *EGR1* MRE-BNIP3 complex formed in the assay. **F** Synthetic MRE RNA oligonucleotide rescues the *EGR1* depletion-induced loss of BNIP3 and its homo-dimerization *in vitro*. After *EGR1* knockdown, the Western assay was performed to detect the abundance of BNIP3 protein and its dimmer. A synthetic RNA oligonucleotide derived from the 3'-end of *EGR1* ntmRNA (SCTL) was used as the control, SP3 group, sMRE group and positive control group (shCTL). Quantitation of relative BNIP3 level was shown in the right panel. Data represents the mean ± SEM of three independent experiments. Significant differences were determined by an unpaired two-tailed *t*-test. ***p < 0.001, ****p < 0.0001.

To clarify how mitochondrial *EGR1* ntmRNA regulates mitophagy, we performed RNA immunoprecipitation (RIP), confirming direct interactions between mitochondrial *EGR1* ntmRNA and mitophagy receptors BNIP3 (**Figure 7A**) and NIX (**Figure S9A**) in HepG2 and SMMC7721 cells. RNA-FISH and immunofluorescence assays further validated mitochondrial colocalization of *EGR1* ntmRNA (green) with BNIP3/NIX proteins (red), significantly reduced upon *EGR1* knockdown (**Figure 7B-C, Figure S9B-E, Figure S10**). These data suggest that mitochondrial *EGR1* ntmRNA regulates mitophagy by interacting with BNIP3/NIX and recruiting them to the mitochondria.

We further used a RIP *in situ* mapping assay 50 to identify the specific fragment of* EGR1* ntmRNA that interacts with BNIP3. After crosslinking, HepG2 cells were lysed and were subjected to a longer sonication to break unbound RNAs. The BNIP3-binding RNAs were immunoprecipitated and were reverse transcribed. The BNIP3-interacting regions were mapped by 21 overlapping PCR reactions (**Figure 7D**, bottom panel). Using this approach, we identified a short 3'-UTR fragment (F19) that showed enriched BNIP3 binding signals (**Figure 7D**, top panel) as compared with the IgG control.

### EGR1 ntmRNA utilizes its 3'-UTR MRE to enhance BNIP3 dimerization

We employed electrophoretic mobility shift assays (EMSA) to confirm that fragment F19 in the *EGR1* 3'-UTR contains a mitophagy receptor-binding element (MRE). A synthetic short MRE oligonucleotide (sMRE) specifically bound BNIP3 protein, forming a distinct complex not seen with a control sequence (sCTL) (**Figure 7E, Figure S11A**).

We further validated the functional role of sMRE in HepG2 cells. *EGR1* knockdown reduced BNIP3 expression and dimerization, while sMRE transfection effectively rescued BNIP3 dimerization, comparable to controls. The control oligonucleotide (sCTL) had no rescuing effect (**Figure 7F**).

### Three-dimensional structure modeling of EGR1 MRE-BNIP3 interaction

To further explore the mechanisms by which *EGR1* ntmRNA regulates BNIP3 dimerization, we utilized automated computational frameworks to predict three-dimensional (3D) structures of *EGR1* MRE and BNIP3 monomer interaction (**Figure S11A-12B**). Notably, with docking analyses to generate models of interactions between the *EGR1* MRE and BNIP3 protein, we demonstrated a high binding affinity model with a docking score of -372.36 (**Figure S11C**). Interaction analyses using PyMOL revealed notable hydrogen bonds between *EGR1* nucleotides A69, C71, and G150 and BNIP3 amino acid residues Ser173, Lys177, Lys176, and Gln139 with hydrogen bonds ranging from 2.2 to 3.3 Å (**Figure S11D**), indicating a strong and specific interaction. Additional simulations assessed the dynamic stability of the interactions, confirming that the identified binding conformation was dynamically stable throughout extended simulation periods, thereby further validating the docking results.

In addition, we also predicted the interaction between the *EGR1* MRE and BNIP3 protein dimer (**Figure S12A-B**). Interestingly, we noted an even stronger binding affinity with a docking score of -423.95 (**Figure S12C**), suggesting a stronger interaction between *EGR*1 MRE and BNIP3 dimer. Interaction analysis using PyMOL identified crucial hydrogen bonds between *EGR1* nucleotides A155, G154, A109, and T110 and amino acids Lys130, Lys135, and Arg139 on the chain A of BNIP3 dimeric protein, with distances ranging from 2.4 to 3.5 Å. Hydrogen bonds were also observed between *EGR1* nucleotides C115, T60, and A56 and residues Lys130, Lys135, and Thr140 on the chain B of BNIP3 dimer, spanning 2.4 to 3.3 Å (**Figure S13A**). These findings indicate a tight binding interaction between *EGR1* ntmRNA and BNIP3 dimer protein.

Finally, we performed an *in vitro* dimerization assay to verify the role of *EGR1* ntmRNA in BNIP3 dimerization (**Figure S13B**). In this assay, mitochondria were isolated from hypoxic HepG2 cells and were incubated with recombinant BNIP3 protein in the presence of *EGR1* ntmRNA. A random RNA (CtRNA) was synthesized as RNA control. BNIP3 dimerization was detected by the Western Blot. Using this *in vitro* system, we verified that *EGR1* ntmRNA enhanced the BNIP3 dimerization in isolated mitochondria (**Figure S13B**, lane 4) as compared with the RNA control (lane 5).

Collectively, these data suggest that *EGR1* ntmRNA may regulate mitophagy initiation and progression in part by coordinating the recruitment and dimerization of BNIP3 mitophagy receptors to the mitochondria.

### EGR1 knockdown affects biological behaviors of hepatoma cells *in vitro*

Mitochondrial dysfunction is closely linked to tumor progression and metastasis. Given the essential role of mitochondrial *EGR1* ntmRNA in mitophagy and mitochondrial function, we assessed the impact of *EGR1* knockdown on malignant behaviors of hepatoma cells. Using HepG2 and SMMC7721 cells, *EGR1* knockdown significantly suppressed cell proliferation, migration, invasion, and colony formation *in vitro* (**Figure S14A-G**). Consistent results were obtained with mitochondria-specific *EGR1* depletion (**Figure S15A-G**). However, transfection with the *EGR1* ntmRNA MRE fragment (sMRE) did not significantly reverse these impaired malignant phenotypes (**Figure S15A-G**). Moreover, *EGR1* knockdown synergistically enhanced the anti-proliferative effect of lenvatinib in HCC cells (**Figure S15G**). However, under the current experimental conditions, this synergy did not translate into significant effects in colony formation or migration/invasion assays. This divergence likely reflects the distinct biological processes assessed: proliferation assays capture acute responses to metabolic stress and cell cycle perturbation, which are more immediately impacted by mitochondrial dysfunction or drug action, whereas colony formation and migration/invasion depend on long-term survival and cytoskeletal remodeling, requiring sustained genetic or epigenetic reprogramming.

Additionally, apoptotic pathway analysis revealed increased expression of apoptotic markers (Cytochrome C, PARP, cleaved PARP, Caspase-3, cleaved Caspase-3, Bax) and decreased anti-apoptotic protein BCL-2 in *EGR1*-knockdown cells (**Figure S14H-I**), indicating that *EGR1* depletion promotes apoptosis in hepatoma cells.

### Knockdown of EGR1 inhibits xenograft tumor growth *in vivo*

**Figure 8:**
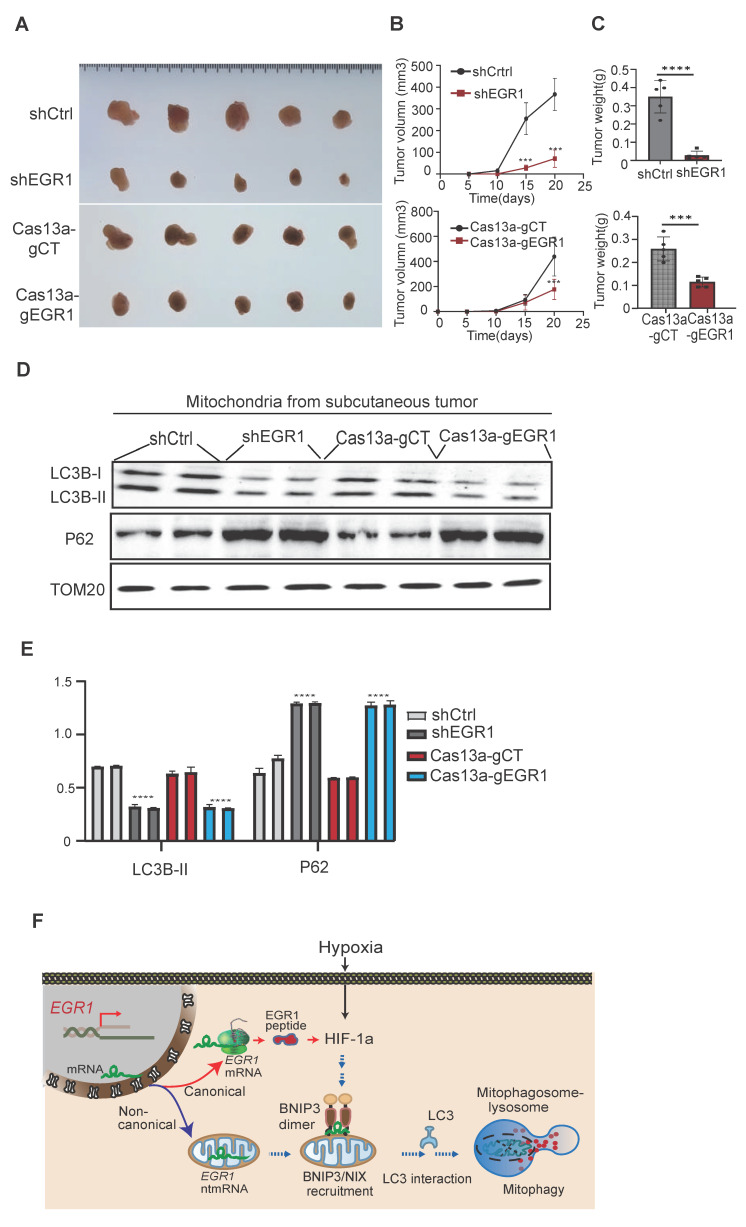
** The role of *EGR1* in xenograft tumor growth in nude mice. A** Photography of subcutaneous xenograft tumors. **B** Tumor growth curves of **s**ubcutaneous tumors in nude mice. **C** Tumor weights of subcutaneous tumors after mice were sacrificed at the end of the study. **D** Expression of LC3B/P62 in subcutaneous tumor mitochondria by Western blot. TOM20 was used as the control. **E** Quantitation of mitochondria LC3B/P62. **F** The model of *EGR1* ntmRNA in the regulation of mitophagy in HCC cells. Hypoxia induces the expression of *EGR1* and HIF-1α. In addition to translating into EGR1 protein to mediate the response to stress, however, some *EGR1* mRNA molecules are aberrantly translocated to mitochondria, where they function as ntmRNA and harness a noncanonical noncoding mechanism to regulate mitophagy in HCC cells. *EGR1* ntmRNA promotes mitophagy through the HIF-1α/BNIP3/NIX pathway. Mechanistically, mitochondrial *EGR1* ntmRNA recruits mitophagy receptor BNIP3 to the mitochondria membrane and coordinate the formation of BNIP3 homo-dimerization. Ubiquitinated proteins are recognized by selective cargo receptors, including p62, Optineurin, and NDP52. Autophagy cargo receptors containing an LC3-interacting region (LIR) bind to LC3 family members and target the autophagosome. Finally, the autophagosome fuses with the lysosome to form mitophagosome-lysosome, which degrades mitochondria. Thus, *EGR1* ntmRNA may regulate hypoxia-induced metabolic reprograming directly in HCC mitochondria via a noncanonical noncoding pathway, in addition to the canonical coding function of EGR1 peptides in ribosomes to initiate the immediate early response to stress. Significant differences were determined by an unpaired two-tailed *t*-test (C) or two-way ANOVA with Tukey's multiple comparison test (B, E). ***P < 0.001, ****P < 0.0001.

To further confirm the role of *EGR1* in tumor growth, we established nude mouse subcutaneous xenograft models using Cas13a-mBN-MLS-mediated mitochondria-specific *EGR1* targeting and shRNA-mediated cellular *EGR1* knockdown. Both approaches significantly inhibited tumor growth rate and reduced tumor volume compared to controls (**Figure 8A-C**), aligning well with the *in vitro* results.

Western blot analysis of tumor tissues revealed decreased LC3B and increased p62 protein levels in *EGR1* knockdown groups (Cas13a-g*EGR1* and sh*EGR1*), confirming that *EGR1* regulates tumor growth via the mitophagy pathway (**Figure 8D-E**). Future studies are needed to compare the synergistic effects of shEGR1 or Cas13a gEGR1 with targeted drugs in HCC.

## Discussion

Little is known about the regulation of mitophagy adaptive response to the hypoxic stress as encountered in the tumor microenvironment. For the first time, we have identified *EGR1* ntmRNA as a non-canonical RNA factor that bridges the hypoxia-mitophagy pathway by coordinating the nucleus-mitochondria-ribosome crosstalk in HCC cells (**Figure 8F**). In the canonical ribosome pathway,* EGR1* mRNA encodes a vital protein factor that is transported back to the nucleus and initiates an immediate early response to the hypoxia stress through the HIF-1α pathway. However, under hypoxic stress, some *EGR1* ntmRNA molecules are translocated to the mitochondria of HCC cells. These translocated mRNA molecules are not translated using mitochondrial translation machinery. Instead, they function as essential non-translating mRNAs to regulate the mitophagy pathway in HCC cells. Mitochondrial knockdown of* EGR1*, whether using LwaCas13a-BN-MLS mitochondrial RNA targeting or shRNA, significantly inhibits mitophagy in HCC cells. Depletion of* EGR1* leads to the accumulation of damaged mitochondria, in parallel with mitochondrial dysfunction, including decreased ATP production, increased ROS production, and decreased mitochondrial membrane potential. Similarly, loss of *EGR1*, either specifically in mitochondria or at the cellular level, inhibits tumor growth both *in vitro* and in nude mice. Importantly, we show that *EGR1* noncoding mRNA molecules utilize their 3'-UTR MRE to recruit mitophagy receptors BNIP3 as well as NIX to mitochondria under hypoxia stimulation. Particularly, *EGR1* ntmRNA coordinates the dimerization of BNIP3 mitophagy receptors on mitochondria, regulating mitophagy initiation and progression (**Figure 8F**). This study provides the first evidence that in addition to its canonical protein coding function, the nuclear genome encoded *EGR1* mRNA may play a non-canonical role in regulating mitophagy through an epigenetic pathway, acting like a long noncoding RNA.

As mitophagy receptors, BNIP3 and NIX function to recruit key autophagy proteins LC3 to the surface of targeted mitochondria for autophagosomal degradation. The N-terminal soluble portion of BNIP3 contains a canonical LC3-interacting region (LIR) motif required for mitophagy. In addition, its C-terminal also contains the transmembrane domain that is essential for the membrane localization and BNIP3 homodimerization required for lysosomal delivery in mitophagy activation 44, 46. Interestingly, we found that mitochondria-specific targeting of* EGR1* ntmRNA inhibited the dimerization of BNIP3. Using RIP-RNase-seq mapping and EMSA assays, we showed that *EGR1* ntmRNA interacted with BNIP3 through a mitophagy receptor-binding element (MRE) in its 3'-UTR. Knockdown of *EGR1* ntmRNA affected mitophagy by reducing BNIP3 and its dimerization in HepG2 cells. However, transfection of a synthetic MRE nucleotide was able to rescue the BNIP3 dimerization. Thus, our data suggests that *EGR1* ntmRNA may use its 3'-UTR MRE to regulate mitophagy by coordinating the recruitment and dimerization of BNIP3 mitophagy receptors to the mitochondria.

Mitophagy is an important biological process by which eukaryotic cells degrade long-lived proteins, misfolded proteins and damaged organelles. Mitophagy consists of several steps, in the early stage, permeability transition occurs after mitochondrial damage, which leads to mitochondrial depolarization and induces mitophagy-related protein activation; and then, autophagosomes wrap around damaged mitochondria, forming mitophagosomes. In the middle stage, mitophagosomes fuse with lysosomes.

Mitophagy flow is a dynamic process, in which these steps appear continuously in the cell. If any step of mitophagy flow is interrupted, mitophagy will not be able to complete its biological function. The activation or inhibition of mitophagy flow can often lead to distinct biological effects. Mitophagy flow blocking in different tissues and organs often leads to disease, including tumors, neurodegenerative diseases, muscle disease 47-49. There are some difficulties in the detection of mitophagic flow. At present, the most widely used method for the detection of mitophagy flow is the detection of LC3BI/II protein by Western blot. However, if there are no drugs that prevent the fusion of the autophagosome to the lysosome and their subsequent degradation, the observed changes in LC3BI/II cannot truly reflect the intracellular mitophagy flow status. For example, we are not sure whether the decrease in LC3B-II expression was caused by either a reduced engulfment of mitochondrial fragments by autolysosomes or an enhanced degradation of mitolysosomes. The detection methods of mitophagy flow are relatively complex, and the single application of a certain method often cannot achieve systematic detection of mitophagy flow, so it is necessary to choose a variety of different experimental methods. In this study, we used the lysosomal degradation inhibitors bafilomycinA1 and pepstatinA+E64d (aloxistatin) which prevents the fusion of the autophagosome to the lysosome and their subsequent degradation, the amount of LC3B-II in the *EGR1* knockdown group was still less than that in the control group, indicating that *EGR1* knock down regulates mitophagy by reducing the formation of autophagosomes rather than increasing the degradation of autophagolysosome. We also analyzed the colocalization of LC3 and the marker of mitochondria (TOM20) by immunofluorescence. Mitochondrial targeted and pH-sensitive fluorescent protein (mito-Kemia) are also used in this study to prove that damaged mitochondria were engulfed by autophaosomes and lysosomes. We demonstrate that *EGR1* mRNA molecules are an essential component in the regulation of mitophagy. Following exposure to hypoxia, HepG2 cells activate the mitophagy pathway to reduce mitochondrial mass thereby limiting ROS generation and maximizing the efficient use of available oxygen. After *EGR1* knockdown, HepG2 cells demonstrate a dramatic accumulation of reactive oxygen species. In the absence of *EGR1*, mitophagy becomes dysregulated in HepG2 cells.

Mitophagy is highly regulated through the tight coordination of multiple signaling pathways 51-54. It is initiated by mitochondrial priming through the Pink1-Parkin signaling pathway or through the mitophagic receptors NIX and BNIP3. A portion of the response can be mediated by receptors that possess C-terminal transmembrane domains localized at the OMM, including BNIP3, NIX and FUNDC1. BNIP3 and NIX are induced by HIF-1 following exposure to hypoxia and are activated by the Foxo3 transcription factor during starvation. Both BNIP3 and NIX contain a typical LC3-interacting region (LIR) motif that interacts directly with LC3/GABARAP and recruits the autophagy machinery to the primed damaged mitochondria 55, 56. FUNDC1 also has an essential role in hypoxia-induced mitophagy via ATG5, but not Beclin 1. Functioning as a mitophagy receptor in mammalian cells, FUNDC1 directly interacts with LC3 and LC3 homologs *via* its LIR domain 57. In the Pink1-Parkin pathway, Pink1 encodes the PTEN-induced kinase, a serine/threonine kinase with a mitochondrial targeting sequence, whereas Parkin is an E3 ubiquitin ligase 56, 58. A subset of outer mitochondrial membrane proteins including VDAC and Mfn 1 and 2 can be ubiquitinated in a Parkin-dependent manner. p62 is recruited to the ubiquitinated mitochondria in CCCP-treated Parkin-positive cells, most likely due to its C-terminal ubiquitin binding domain. Upon mitochondrial membrane depolarization, proteasomes can be recruited to the mitochondria in a Parkin-dependent manner, which leads to the degradation of the mitochondrial outer membrane and the intermembrane space proteins, but not the inner-membrane and matrix proteins. In this study, we showed that *EGR1* regulates mitophagy primarily through the BNIP3/NIX pathway. In this pathway, *EGR1* noncoding mRNA molecules interact with both BNIP3 and NIX to help coordinate the recruitment of these mitophagy receptors to the mitochondria membrane under hypoxia stimulation. Notably, we found that the overexpression of BNIP3 or NIX in *EGR1* knockdown cell lines could not revert the repression of mitophagy. Therefore, the mitophagy promoting effect of *EGR1* may not depend entirely on BNIP3 or NIX. The other regulatory pathways only account for a small portion of mitophagy activity in *EGR1*-knockdown cells.

*EGR1* encodes a nuclear transcriptional regulatory protein belonging to the C2H2-type zinc finger-containing EGR family. This zinc-finger transcription factor binds to the conserved DNA motif sequence GCG(T/G)GGGCG in the promoters of target genes and coordinates the response to a variety of stress stimuli 59, 60. Like other coding mRNAs, *EGR1* mRNAs are present in the cytoplasm where they are translated into EGR1 peptides on ribosomes. However, using ribosome RNA sequencing and quantitative PCR as well as RNA-FISH confirmation, we now show that *EGR1* mRNA can be translocated into the mitochondria of HepG2 cells where it may play a non-canonical role in regulating the function of mitochondria through a heretofore unknown “non-translating mRNA” (ntmRNA) pathway. This function represents an entirely new role for an mRNA molecule and suggests that cancer cells can hijack RNAs for novel functions. Interestingly, the transfection of synthetic *EGR1* ntmRNA MRE fragments (sMRE) did not significantly rescue the impaired malignant phenotypes observed after mitochondrial *EGR1* knockdown (**Figure S15A-G**). This limited functional recovery might be due to technical challenges in efficiently delivering sMRE fragments specifically to mitochondria, rather than an indication of their biological insignificance.

It should be noted that *EGR1* has been reported to exhibit both tumor-suppressive and pro-oncogenic properties in HCC, a duality largely shaped by the cellular signaling context, including microenvironmental cues such as hypoxia. In our study, we observed a marked upregulation of *EGR1* mRNA under hypoxic conditions in HCC cells, supporting its context-dependent regulatory function during tumor adaptation to stress.

In summary, *EGR1* is an essential coordinator in response to a variety of stress stimuli, including hypoxia, oxidative stress, DNA damage, ischemia, physical forces, growth factors, cytokines, and tissue injury. The specific role of *EGR1* in tumors, including HCC, remains elusive 34, 61-67. The findings from this study suggest that* EGR1* may play an oncogenic role in HCC cells by coordinating the nucleus-mitochondria-ribosome crosstalk. Upon exposure to hypoxia, the *EGR1* gene is activated in the nucleus and encodes *EGR1* mRNA molecules. Through the canonical ribosome pathway, *EGR1* mRNAs are translated into EGR1 peptides that initiate mitophagy through HIF-1α in the nucleus. However, some ntmRNA are translocated into mitochondria, where they epigenetically regulate mitophagy through the BNIP3/NIX pathway. Mechanistically, the mitochondria-localized *EGR1* ntmRNA utilizes a 3'-UTR MRE element to coordinate the recruitment and dimerization process of mitophagy receptor BNIP3 to mitochondria. Our study thus suggests a novel non-canonical genetic pathway where *EGR1* ntmRNA has a noncoding function in mitochondria. Hence,* EGR1* plays an essential role in maintaining a healthy mitochondrial network through the tight coordination of the genetic messages within the nucleus-ribosome-mitochondria axis.

## Materials and Methods

### Cell culture

HepG2, SMMC7721, THLE-2, THLE-3, HHL-5, and 293T cells were obtained from ATCC or BLUEBIO. Cells were cultured in Dulbecco's Modified Eagle Medium (DMEM, Thermo Fisher) containing 10% FBS and 1% penicillin/streptomycin at 37°C with 5% CO2. HL7702 cells were maintained similarly in RPMI-1640.

### Animal studies

Animal procedures were approved by The First Hospital of Jilin University ethics committee. Twenty 5-week-old female BALB/c nude mice were divided into four groups (n=5/group): Cas13a-gCT control, Cas13a-gEGR1, shCtrl, and shEGR1. HepG2 cells (5 × 10^5 in 100 μl PBS) were subcutaneously injected. Tumor volumes were measured every 5 days (volume = length × width²/2). Mice were sacrificed on day 21 for tumor analysis.

### Hypoxia treatment

Cells were incubated in a modular hypoxia chamber (Billups-Rothenberg) flushed with hypoxic gas (1% O2, 5% CO2, 94% N2) at 5 L/min.

### Mitochondrial isolation and RNA sequencing

Mitochondria were isolated using a modified Qproteome Mitochondria Isolation Kit (QIAGEN). RNase A treatment eliminated cytoplasmic RNAs. RNA extraction from mitochondria and whole cells was performed using TRIzol (Invitrogen). cDNA synthesis followed DNase I digestion using M-MLV reverse transcriptase. Mitochondrial purity was confirmed by qPCR (COX2 positive, U6 negative). RNA sequencing used HiSeq4000 (Illumina), selecting genes with fold-change >2, p<0.05, and FPKM>50.

### Quantitative real-time PCR (qPCR)

Real-time PCR utilized FastStart SYBR Green Master Mix (Sigma) on an ABI Prism 7900HT system. Gene expression was normalized against mitochondrial COX2 or cellular β-actin. Primer sequences are listed in Table S1.

### RNA-FISH assay

RNA-FISH utilized antisense DNA probes labeled with digoxigenin. RNAs in mitochondria were detected with anti-digoxigenin-fluorescein and visualized with MitoTracker Red CMXRos.

### Immunofluorescence (IF)

Cells were fixed (4% paraformaldehyde), permeabilized (0.5% Triton X-100), and blocked (1% BSA). Samples were incubated with primary antibodies (EGR1, TOM20) and Alexa Fluor-conjugated secondary antibodies. Hoechst 33258 stained nuclei, and images were acquired with a Zeiss AxioCam.

### RNA immunoprecipitation (RIP)

RIP assays used the Magna RIP Kit (EMD Millipore). Lysates from HepG2 and SMMC7721 cells were immunoprecipitated with anti-BNIP3/NIX antibodies. RNA was isolated and detected by RT-qPCR. Rabbit IgG was the negative control.

### Mitochondria-specific RNA knockdown

Cas13a-mBN-MLS system targeted mitochondrial *EGR1* ntmRNA. Lentiviruses carried mitochondria-localized Cas13a fused with mutant Barnase (mBN) and MLS from COX8A. *EGR1*-targeting gRNAs and negative controls were cloned into lentiviral vectors. Transfected cells underwent puromycin selection.

### Cell transfection

Lentiviral shRNAs, siRNAs, and plasmids for *EGR1*, BNIP3, NIX, and HIF-1α were transfected using Lipofectamine 2000 (Invitrogen). shRNAs targeted *EGR1*, with random sequences as controls. Selection was by puromycin.

### Mitochondrial function assays

ATP, mitochondrial superoxide, mitochondrial membrane potential (JC-1), ROS (DCFDA, MitoSOX), OCR, and ECAR assays followed manufacturers' protocols (Beyotime, Abcam, Agilent Seahorse XF24e).

### Mitophagy and autophagic flux assays

Mitophagy was assessed using mt-Keima and mRFP-GFP-LC3 lentiviruses. Cells were treated with CCCP or hypoxia, and fluorescence signals were analyzed via confocal microscopy.

### Electrophoretic mobility shift assay (EMSA)

EMSA utilized the LightShift RNA EMSA Kit (Thermo Scientific). RNA-protein binding was detected with chemiluminescence.

### *In vitro* BNIP3 dimerization

Isolated mitochondria were incubated with *EGR1* ntmRNA or control RNA and recombinant BNIP3 protein. BNIP3 dimerization was assessed by Western blot.

### Structural prediction of RNA-protein interactions

Computational modeling used 3dRNA/DNA, 3dRPC, ASPDock, and HDOCK. Models were refined with DISCOVERY STUDIO 2019, and interactions were visualized using PyMOL.

### Western blot analysis

Protein lysates were resolved by SDS-PAGE, transferred to PVDF membranes, blocked, and incubated with primary/secondary antibodies. Chemiluminescence detection was quantified with ImageJ. Antibodies are listed in Table S2.

### Transmission electron microscopy

Cells were fixed (4% glutaraldehyde), post-fixed (2% osmium tetroxide), dehydrated, embedded in Spurr resin, sectioned, stained (uranyl acetate/lead citrate), and observed by TEM (HITACHI H-7650).

### Cell proliferation, colony formation, migration, and invasion

Proliferation (CCK-8), colony formation, Transwell invasion, and wound-healing assays were performed as described, quantifying cell growth, clonogenicity, invasion, and migration abilities.

### Statistical analysis

Data presented as mean ± SD. Differences assessed by Student's t-test or ANOVA (Bonferroni correction). Significance set at p<0.05, analyzed using GraphPad Prism 7.

## Supplementary Material

Supplementary figures and tables.

## Abbreviations

EGR1: early growth response 1
HCC: hepatocellular carcinoma
LwaCas13a-BN-MLS: the LwaCas13a-Barnase mutant-mitochondrial localization signal peptide mitochondrial RNA targeting system
ROS: reactive oxygen species
BLIP3: BCL2/adenovirus E1B 19 kDa protein-interacting protein 3
FISH: fluorescence *in situ* hybridization
TEM: transmission electron microscope
FUNDC1: FUN14 domain containing 1
RIP: RNA chromatin immunoprecipitation
OMM: outer mitochondrial membrane
Q-PCR: quantitative real-time PCR
IF: immunofluorescence
OCR: oxygen consumption rate
ECAR: extracellular acidification rate
EMSA: electrophoretic mobility shift assay
MRE: mitophagy receptor-binding element
