# Supplementary material for: Mitochondria-specific targeting of noncanonical EGR1 ntmRNA-coordinated mitophagy receptor BNIP3 homodimerization disrupts mitochondrial metabolism and suppresses hepatocellular carcinoma growth in vitro and in vivo
Source: Theranostics. 2026 Jan 1;16(4):1681–700. doi: 10.7150/thno.117745 (PMC12680496; doi:10.7150/thno.117745)
Supplement: Supplementary file 1 — Supplementary figures and tables. [file thnov16p1681s1.pdf]

Mitochondria-specific targeting of noncanonical *EGR1* ntmRNA-coordinated mitophagy receptor BNIP3 homodimerization disrupts mitochondrial metabolism and suppresses hepatocellular carcinoma growth *in vitro* and *in vivo*

Yan Li[1], Lei Zhou[1], Mengmeng Liu[1], Hui Li[1], Ying Meng[1], Xue Wen[1], Yijing Zhao[1,2], Shanshan Liu[1], Zi Yan[1,2], Changhao Fu[1,2], Shan Zong[1], Wei Li[1], Andrew R. Hoffman[2], Jiuwei Cui[1*], Ji-Fan Hu[1,2*]

[1] Cancer Center, First Hospital of Jilin University, Changchun, Jilin 130061, China

[2] Stanford University Medical School, VA Palo Alto Health Care System, Palo Alto, CA 94304, USA

Supplemental tables

Table S1. Oligonucleotides used for PCR and *EGR1* knockdown

Primer name	Sequences (5’-3’)
EGR1	AGCAGCACCTTCAACCCTCAG
	GTAACTGGTCTCCACCAGCA
U6	GTGCTCGCTTCGGCAGCACATATAC
	ATATGGAACGCTTCACGAATTTGCG
Cox2	ATGGCACATGCAGCGCAAGTAGGTC
	GTTAGGAAAAGGGCATACAGGAC
β-actin	CAGGTCATCACCATTGGCAATGAGC
	CGGATGTCCACGTCACACTTCATGA
COX1	CGCTATCCCCACCGGCGTCAAAG
	GTCGTGTAGTACGATGTCTAGTG
MT-CYBT	GACAGACCTAGTTCAATGAATC
	ATAGGGCAAGGACGCCTCCTAG
MT-ND1	GCATACCCCCGATTCCGCTA
	TGAGGGGGAATGCTGGAGAT
MT-ND2	CATGCTAGCTTTTATTCCAGTTC
	GTTATGGTTCATTGTCCGGAGAGT
MT-ND3	CGAGTGCGGCTTCGACCCTA
	AGGGGTAAAAGGAGGGCAATTTC
U6 promoter	GCATATACGATACAAGGCTGTTA
	GGTGTTTCGTCCTTTCCACAAGAT
SMRE	TAATACGACTCACTATAGGGCCAATGGTGATCCTC
	ATGTGAGAGTACGGTCAAGCAG
SP3	TAATACGACTCACTATAGGGCCAATGGTGATCCTC
	GAACCGAAGCTCAGCTCAGC
SCTL	GACCGTACTCTCACATGTGGC
	CCCAATCGCAGCTACTTTATTTTCG
HA tag	GTCGTGACGCGGATCCTCTAGACGCCACCATGTACCCATACGACGTCCCAGACTACGCT
	GCTGATGCCGTCGACCTTGGTCACTTTACCAGCGTAGTCTGGGACGTCGTA
shEGR1-1	CGTCCCCGGTTACTACCTCTTAT
shEGR1-2	ACTCTTGATGTGAAGATAATTTGCA
shCT	GCAGCAACTGGACACGTGATCTTAA
Cas13a gRNA	ATAAGAGGTAGTAACCGGGGACGGGTAA
Cas13a gCT	GTTGCAGTAGATCTTCGACTAGATCTAC
siEGR1	CGTCCCCGGTTACTACCTCTUU

siCtrl (sense)	UUCUCCGAACGUGUCACGUTT
siCtrl (antisense)	ACGUGACACGUUCGGAGAATT

Table S2. Antibodies

Antibodies	Company	Dilution	MW(kDa)
EGR1 (15F7) Rabbit mAb	Cell Signaling Technology	1:1000	75
OPA1 (D6U6N) Rabbit mAb	Cell Signaling Technology	1:1000	80-100
Mitofusin-1 (D6E2S) Rabbit mAb	Cell Signaling Technology	1:1000	82
Mitofusin-2 (D2D10) Rabbit mAb	Cell Signaling Technology	1:1000	80
Phospho-DRP1(Ser616) Rabbit mAb	Cell Signaling Technology	1:1000	78-82
Phospho-DRP1(Ser637) Rabbit mAb	Cell Signaling Technology	1:1000	78-82
LC3B (D11) XP® Rabbit mAb	Cell Signaling Technology	1:1000	14,16
Phospho-SQSTM1/p62 (Ser349) (E7M1A) Rabbit mAb	Cell Signaling Technology	1:1000	62
Optineurin (E4P8C) Rabbit mAb	Cell Signaling Technology	1:1000	75
NDP52 (D1E4A) Rabbit mAb	Cell Signaling Technology	1:1000	52,60
MFF(E5W4F) Rabbit mAb	Cell signaling Technology	1:1000	25,27,30,35
HIF-1α (D1S7W) XP® Rabbit mAb	Cell Signaling Technology	1:1000	120
BNIP3 (D7U1T) Rabbit mAb	Cell Signaling Technology	1:1000	22-28, 50-55
BNIP3L/Nix (D4R4B) Rabbit mAb	Cell Signaling Technology	1:1000	38,76
Parkin (Prk8) Mouse mAb	Cell Signaling Technology	1:1000	50
PINK1 (D8G3) Rabbit mAb	Cell Signaling Technology	1:1000	60,50
Bcl-2 (124) Mouse mAb	Cell Signaling Technology	1:1000	26
Cytochrome c (136F3) Rabbit mAb	Cell Signaling Technology	1:1000	14
Caspase-3(D3R6Y) Rabbit mAb	Cell Signaling Technology	1:1000	17, 19, 35
PARP Antibody 9542	Cell Signaling	1:1000	89,116

	Technology		
Cleaved PARP(Asp214)	Cell Signaling Technology	1:1000	89
Cleaved Caspase-3(Asp175)	Cell Signaling Technology	1:1000	17,19
Bax	Cell Signaling Technology	1:1000	20
FUNDC1 Polyclonal Antibody	Thermo fisher	1:1000	18
Anti-MTCO1 antibody	Abcam	1:800	37
Recombinant Anti-TOMM20 antibody	Abcam	1:1000	16
COX IV (3E11) Rabbit mAb	Cell Signaling Technology	1:1000	17
Anti-beta Actin antibody	Abcam	1 µg/ml	42
Anti-mouse IgG, HRP-linked Antibody	Cell Signaling Technology	1:2000	/
Anti-rabbit IgG, HRP-linked Antibody	Cell Signaling Technology	1:2000	/

Supplemental figures

Figure S1. Mitochondria-specific RNA targeting using the LwaCas13a-mBN-MLS system.

A LwaCas13a-mBN-MLS targeting and control vectors. pU6: RNA polymerase III U6 promoter; gEGR1: Cas13a guiding RNAs that target the EGR1 ntmRNA; T5: the TTTTT termination signal of RNA polymerase III; pEF1: the EF1-alpha promoter; HA: HA tag peptide, H-Tyr-Pro-Tyr-Asp-Val-Pro-Asp-Tyr-Ala-OH (YPYDVPDYA), used for tracking Cas13a; Cas13a: the catalytically inactive CRISPR Cas13a; MLS: mitochondria-localization signal peptide derived from COX8A, which helps the specific localization of the Cas13a-mBN in the mitochondria; gCT: a random gRNA control.

B Mitochondria-specific targeting of *EGR1* ntmRNA. In this system, Cas13a-gRNA provides the RNA targeting specificity. MLS provides the specific localization of the Cas13a-mBN in the mitochondria. The potent RNase activity of mBN efficiently degrades *EGR1* ntmRNA in the mitochondria. Working together, this system effectively reduces the levels of *EGR1* ntmRNA in the mitochondria, thereby allowing for the study of mitochondrial RNA function and its impact on cellular processes.

C The heatmap displays the top 10 differentially enriched RNAs in the mitochondria of HepG2 and HL7702 cells, based on RNA-seq analysis. Expression levels are represented by a color scale, with red indicating higher expression and white indicating lower expression. The color bar on the right shows the relative expression intensity, with yellow and blue corresponding to HepG2 and HL7702 cells, respectively. EGR1 exhibits the most significant differential expression between the two cell lines, supporting its selection for further investigation.

Figure S2. Mitochondrial enrichment of *EGR1* ntmRNA in hepatoma cells.

A Quantification of *EGR1* ntmRNA in isolated HepG2 mitochondria by qPCR. The U6 RNA was used as quality control for isolated mitochondria. The Ct value of *EGR1* was normalized over that of the mitochondria-encoded COX2 and set as 1 for comparison.

B Quality check of isolated mitochondrial RNAs. The nuclear genome-encoded U6 RNA was used as the control. At the whole-cell level, COX2, *EGR1* and U6 RNA were detected (left panel). Both *EGR1* and mitochondrial control COX2 were detected in isolated HepG2 mitochondria. No nuclear U6 RNA was

detected in mitochondrial RNA samples (right panel).

C RNA-FISH detection of *EGR1* ntmRNA in Hepatoma SMMC7722 cells. The nuclei were stained with DAPI (blue). *EGR1* ntmRNA was probed by Dig-labeled single-stranded DNA probes (green). Mitochondria were labeled with MitoTracker (red). The nuclear-encoded *U6* RNA was used as a negative control, and the mitochondria-encoded *COX2* served as a positive control.

D Colocalization quantitation of *EGR1* ntmRNA and MitoTracker using Scatter plot. Pearson's correlation coefficient (PCC) was calculated for the entire image (Rtotal) and the pixels above thresholds (Rcoloc). 1: perfect correlation; -1: completely excluded; 0: random relationship.

E Total *EGR1* RNA levels between normal hepatocytes and hepatocellular carcinoma (HCC) Cells. HepG2 and SMMC7721: hepatoma cells; THLE-2, THLE-3 and HHL-5: normal hepatic cells; 293T: normal human embryonic kidney cells. The Ct values were normalized over that of β-Actin and then standardized by setting 293T as 1 for comparison.

Data represents the mean ± SEM of three independent experiments. Significant differences were determined by an unpaired two-tailed *t*-test. **** $p < 0.0001$.

Figure S3. The mitochondria-enriched *EGR1* mRNA is not translated in the mitochondria of hepatoma cells.

A The nucleus-mitochondria-ribosome crosstalk model. LncRNAs can shuttle between nuclei and mitochondria and function as epigenetic messengers to coordinate cell metabolism. The nuclear-encoded lncRNAs that translocate to mitochondria are so-called anterograde signals. On the other hand, the mitochondria-encoded lncRNAs that shuttle to the nucleus, function as retrograde signals. It is not clear if *EGR1* mRNA molecules in the HCC mitochondria function as an anterograde signal or translate to EGR1 peptides as they do in the ribosome. Dashed line: unproven crosstalk pathways.

B Western bolt of EGR1 protein levels in total cells and isolated mitochondria from HepG2 cells. β-ACTIN and TOM20 were used as the controls. No EGR1 proteins were detected in isolated mitochondria, suggesting an anterograde messenger role of *EGR1* mRNA as a non-translating mRNA (ntmRNA) in

HCC cells.

C Immunofluorescence staining of EGR1 protein under hypoxia 0h and 24h. Nuclei were stained with DAPI (blue) and EGR1 protein was immunostained by the anti-EGR1 antibody (green). Mitochondria were labeled with MitoTracker (purple). No EGR1 protein was detected in mitochondria. Scale bar: 10μm.

Figure S4. *EGR1* knockdown affects mitochondrial functions in HepG2 cells.

A Schematic diagram of *EGR1* shRNA vectors.

B Knockdown of *EGR1* by shRNA lentiviruses in HepG2 cells. shEGR1-1, shEGR1-2: two *EGR1* shRNAs; shCtrl: random shRNA control. *EGR1* was quantitated by qPCR, and the Ct value was normalized over that of β-Actin and then standardized by setting untreated HepG2 cells as 1.

C ATP synthesis in shCtrl and shEGR1-treated HepG2 cells.

D Mitochondrial membrane potential in shCtrl and shEGR1-treated HepG2 cells following the CCCP treatment.

Data represents the mean ± SEM and are representative of three independent experiments. Significant differences were determined by an unpaired two-tailed *t*-test. ** $p < 0.01$, *** $p < 0.001$, **** $p < 0.0001$.

Figure S5. *EGR1* Knockdown interferes with mitochondrial function in SMMC7721 cells.

A Knockdown of *EGR1* by shRNA lentiviruses in SMMC7721 cells. sh*EGR1*-1, sh*EGR1*-2: two *EGR1* shRNAs; shCtrl: random shRNA control. *EGR1* was quantified by qPCR, and the Ct value was normalized over that of β-Actin and then standardized by setting untreated SMMC7721 cells as 1.

B ATP synthesis in shCtrl and shEGR1-treated SMMC7721 cells.

C Mitochondrial membrane potential in shCtrl and sh*EGR1*-treated SMMC7721 cells with or without CCCP treatment.

D Rescue assay in SMMC7721 cells. siEGR1-treated cells were rescued by transfection of *EGR1* plasmid DNA. Rescue cells were cultured under hypoxic conditions for 12h and were used for ROS fluorescence staining using the ROS probe DCFDA/H2DCFDA (green). Mitochondria were tracked by MitoTracker

Deep Red (MTDR). The yellow color indicates accumulated ROS within the mitochondria. Quantitative changes in the ROS green fluorescence intensity were analyzed by Image J. Scale bar: 20μm.

E Cellular ROS in SMMC7721 cells. ROS was examined using DCFDA probe followed by flow cytometry in the siCtrl (yellow), siEGR1 (blue) and siEGR1-rescue (pink) Groups.

Data represents the mean ± SEM and are representative of three independent experiments. Significant differences were determined by an unpaired two-tailed *t*-test. ** $p < 0.01$, *** $p < 0.001$, **** $p < 0.0001$.

Figure S6. Cas13a-mBN-MLS targeting of *EGR1* ntmRNA affects mitochondria mass in HepG2 cells.

A Immunofluorescent quantification of TOM20. HepG2 cells were transfected with lentiviruses carrying the Cas13a-mBN-MLS system and were incubated under normoxic and hypoxic conditions. Cas13a-gCT: random Cas13a gRNA; Cas13a-gEGR1: *EGR1* Cas13a gRNA1. Mitochondria were tracked by immunostaining of TOM20 (red). Nuclei were stained with DAPI (blue). In response to hypoxia stress, cells eliminated mitochondria using the mitophagy pathway. Mitochondria-specific knockdown of *EGR1* inhibited mitophagy.

B Western blot of COX4 in Cas13a-mBN-MLS-treated cells. Cells were collected for Western blotting after exposure to hypoxia for 0h,12h, and 24h.

C Quantification for TOM20 level after hypoxia treatment in Figure A. (40 cells were counted per group).

D Quantification of COX4 Western blot in Figure B.

Data represents the mean ± SEM of three independent experiments. Significant differences were determined by an unpaired two-tailed t-test (C) or two-way ANOVA with Tukey's multiple comparison test (D). * $p < 0.05$, ** $p < 0.01$, **** $p < 0.0001$.

Figure S7. Mitochondria mass in shEGR1 knockdown HepG2 cells.

A Immunofluorescent staining of TOM20 in shEGR1-treated cells under normoxic and hypoxic conditions.

Nuclei were stained with DAPI (blue), and TOM20 was immunostained in red.

B Quantification for TOM20 level after hypoxia treatment in Figure A. (40 cells were counted per group).

C Western blot of COX4 in shEGR1-rescue cells under normoxic and hypoxic conditions. β-Actin was used as the control.

D Quantification of COX4 in Figure C.

Data represents the mean ± SEM of three independent experiments. Significant differences were determined by an unpaired two-tailed t-test (C) and two-way ANOVA with Tukey's multiple comparison test (D). * $p < 0.05$,** $p < 0.01$.

Figure S8. *EGR1* ntmRNA is required for hypoxia-induced mitochondrial fission and mitochondrial fusion.

A Western blot measurement of mitochondria fission and fusion proteins. After hypoxia treatment for 0h, 12h and 24h, HepG2 cells were collected for Western blotting. β-ACTIN was used as the control.

B Quantification of Western blot for mitochondria fission and fusion proteins. The 0h group was set as 1 for comparison.

Data represents the mean ± SEM of three independent experiments. Significant differences were determined by two-way ANOVA with Tukey's multiple comparison test. * $p < 0.05$, ** $p < 0.01$, *** $p < 0.001$, **** $p < 0.0001$.

Figure S9. *EGR1* ntmRNA interacts with the mitophagy receptor NIX in mitochondria.

A *EGR1* ntmRNA-NIX interaction by the RIP assay. HepG2 and SMMC7721 cells were collected after exposure to hypoxia for 0h and 24h, and the RIP assay was used to detect the interaction of *EGR1* ntmRNA with the mitophagy receptor NIX. Ct value was normalized over that of β-ACTIN and then standardized by setting IgG as 1.

B Colocalization of *EGR1* ntmRNA with mitochondrial NIX in HepG2 cells by RNA-FISH and immunofluorescent staining. The nuclei were stained with DAPI (blue), and mitochondria were labeled with Mito-Tracker (yellow). *EGR1* ntmRNA was probed with Dig-labeled single-stranded DNA probes and detected with FITC-coupled anti-dig antibody (green). Anti-NIX antibody was used to label NIX protein (Red). The overlapping part of *EGR1* mRNA with NIX proteins appeared in yellow. Scale bar: 10μm.

C Colocalization of *EGR1* ntmRNA with mitochondrial NIX in SMMC7721 cells. Scale bar: 10μm.

D Quantitation of *EGR1* ntmRNA-NIX interaction yellow puncta numbers in HepG2 cells (fluorescent scanning of Figure B).

E Quantitation of *EGR1* ntmRNA-NIX interaction yellow puncta numbers in SMMC7721 cells (fluorescent scanning of Figure C).

Data represents the mean ± SEM of four independent experiments. Significant differences were determined by an unpaired two-tailed t-test. **$p < 0.01$, ***$p < 0.001$.

Figure S10. *EGR1* ntmRNA interacts with mitochondrial BNIP3 in SMMC7721 cells.

A Colocalization of *EGR1* ntmRNA with mitochondrial BNIP3 in SMMC7721 cells by RNA-FISH and immunofluorescent staining. The nuclei were stained with DAPI (blue) and mitochondria were labeled with Mito-Tracker (yellow). *EGR1* ntmRNA was probed with Dig-labeled single-stranded DNA probes and detected with FITC-coupled anti-dig antibody (green). Anti-BNIP3 antibodies were used to label BNIP3 proteins (Red). The overlapping portion of *EGR1* ntmRNA with BNIP3 proteins appeared in yellow. Scale bar: 10μm.

B Quantitation of *EGR1* ntmRNA-BNIP3 interaction yellow puncta numbers in SMMC7721 cells (fluorescent scanning of Figure A) ($n = 4$). Data represents the mean ± SEM of three independent experiments. Significant differences were determined by an unpaired two-tailed *t*-test. *** $p < 0.001$.

Figure S11. Interaction model construction between *EGR1* ntmRNA MRE and BNIP3 monomer.

A *EGR1* MRE and BNIP3 monomer binding nucleotides. Specific MRE nucleotides (A69, C71, and G150) that interact with BNIP3 monomer are marked in red.

B Model construction using PYMOL. After docking, the top 10 binding models were extracted.

The scores of the top 10 binding models.

C Molecular model of the interaction between *EGR1* ntmRNA MRE and BNIP3 monomer. The size and location of the interaction is visualized with PyMOL. The insert shows the specific interaction sites and hydrogen bonds ranging from 2.2 to 3.3 Å between EGR1 MRE nucleotides (A69, C71, G150) and BNIP3 amino acid residues (Ser173, Lys177, Lys176, Gln139).

Figure S12. Interaction analysis between *EGR1* ntmRNA MRE and BNIP3 dimer.

A *EGR1* MRE and BNIP3 dimer binding nucleotides. The *EGR1* MRE nucleotides that interact with BNIP3.

B dimer chains A and B are shown in blue and purple, respectively.

Model construction using PYMOL. After docking, the top 10 binding models were extracted.

C The scores of the top 10 binding models between *EGR1* ntmRNA MRE and BNIP3 dimer.

Figure S13. *EGR1* ntmRNA induces BNIP3 dimerization.

A The intera3 dimeric protein chain A amino acids (Lys130, Lys135, Arg139), as well as the hydrogen bonds between *EGR1* MRE nucleotides (C115, T60, DA56) and BNIP3 dimer chain B residues (Lys130, Lys135, Thr140).

B *EGR1* ntmRNA MRE induces BNIP3 dimerization on isolated mitochondria. Top panel: Schematic diagram of the BNIP3 dimerization assay. Mitochondria from hypoxic HepG2 cells were incubated with BNIP3 protein in the binding buffer containing *EGR1* ntmRNA. BNIP3 dimerization was detected by the Western Blot. Bottom panel: BNIP3 dimerization. After in vitro incubation, BNIP3 dimerization was determined by the Western Blot. A random RNA (CtRNA) was used as the RNA control in the assay.

Figure S14. ***EGR1* affects malignant phenotypes of hepatocellular carcinoma cells in mitophagy dependent manner.**

A Cell invasion. Transwell assay was used to assess cell invasion in shEGR1-treated HepG2 cells and SMMC7721 cells.

B Quantitation of the Transwell assay. Five randomly selected fields were photographed and counted per well.

C Colony formation assays of shEGR1-treated HepG2 cells and SMMC7721 cells in 6-well plates.

D Quantitation of the colony formation assays by ImageJ.

E Cell Migration by the wound healing assay in shEGR1-treated HepG2 cells and SMMC7721 cells.

F The wound closed area (%) was measured by ImageJ.

G Cell proliferation assay. Cell proliferation was measured by the CCK-8 kit in shEGR1-treated HepG2 cells and SMMC7721 cells and was calculated as the slope ratio of the growth OD curves.

H Expression of apoptotic proteins by Western blot. β-ACTIN was used as the control.

I Quantitation of apoptotic proteins.

Data represents the mean ± SEM of three independent experiments. Significant differences were determined by an unpaired two-tailed *t*-test or two-way ANOVA with Tukey's multiple comparison test. *$p < 0.05$, **$p<0.01$, ***$p < 0.001$, ****$p < 0.0001$.

Figure S15. **Mitochondria-targeted knockdown of *EGR1* affects HCC phenotypes.**

A Transwell invasion assays were performed to assess cell invasion capacity in the following groups: control group, mitochondria-targeted *EGR1* knockdown group, and mitochondria-targeted *EGR1* knockdown combined with MRE fragment rescue group in HepG2 cells and SMMC7721 cells.

B Quantitation of the Transwell assay. Five randomly selected fields were photographed and counted per well.

C Colony formation assays were performed to assess cell invasion capacity in the following groups: control group, mitochondria-targeted *EGR1* knockdown group, and mitochondria-targeted *EGR1* knockdown combined with MRE fragment rescue group in HepG2 cells and SMMC7721 cells.

D Quantitation of the colony formation assays by ImageJ.

E The wound healing assay was performed to assess cell migration capacity in the following groups: control group, mitochondria-targeted *EGR1* knockdown group, and mitochondria-targeted *EGR1* knockdown combined with MRE fragment rescue group in HepG2 cells and SMMC7721 cells.

F The wound closed area (%) was measured by ImageJ.

G Cell proliferation was measured by the CCK-8 OD slopes in control group, mitochondria-targeted *EGR1* knockdown group, and mitochondria-targeted *EGR1* knockdown combined with MRE fragment rescue group in HepG2 cells and SMMC7721 cells.

H Expression of apoptotic proteins by Western blot. β-ACTIN was used as the control.

I Quantitation of apoptotic proteins.

Data represents the mean ± SEM of three independent experiments. Significant differences were determined by an unpaired two-tailed *t*-test or two-way ANOVA with Tukey's multiple comparison test. *$p < 0.05$, **$p<0.01$, ***$p < 0.001$, ****$p < 0.0001$.

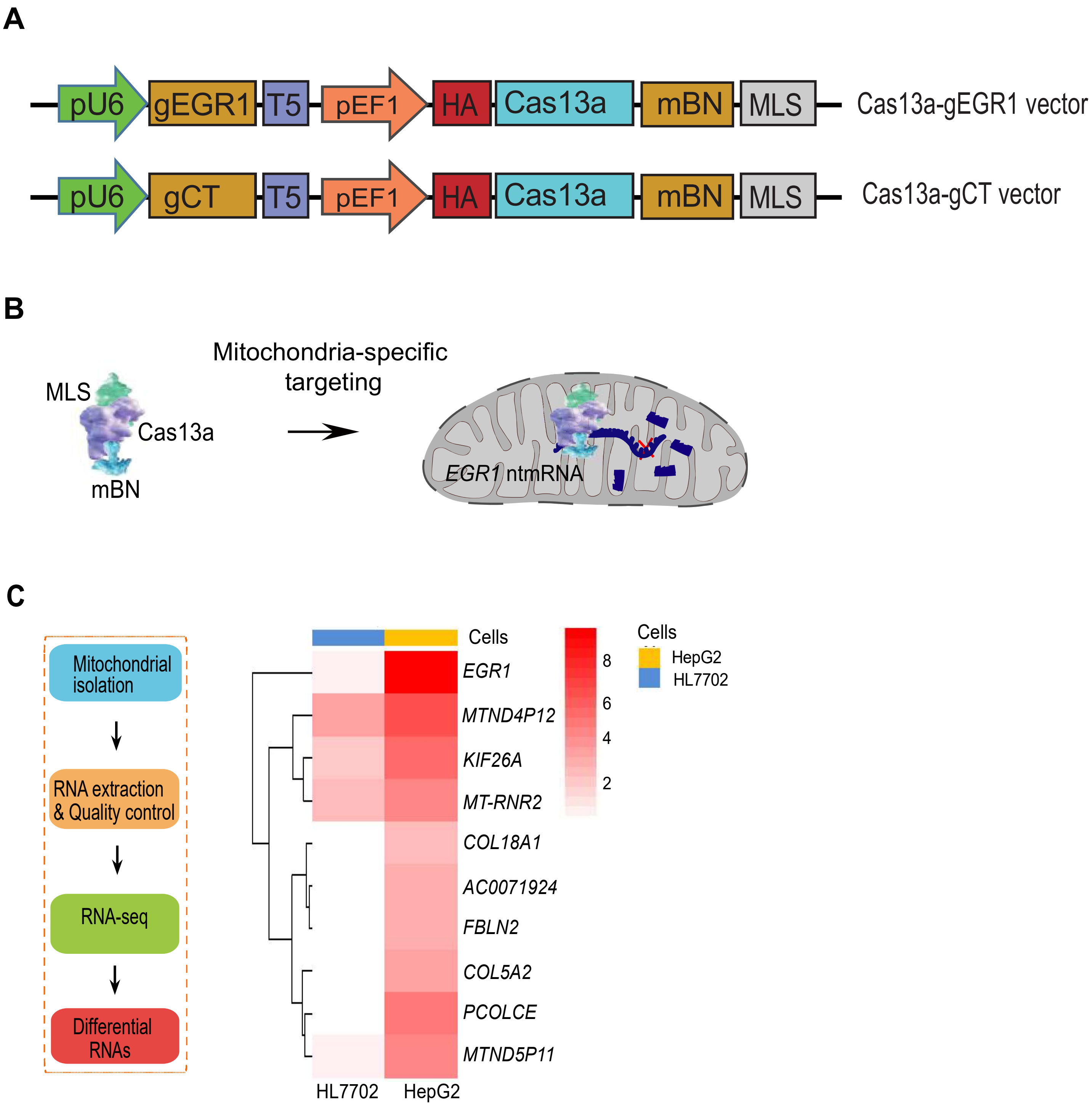
A
pU6
gEGR1
T5
pEF1
HA
Cas13a
mBN
MLS
Cas13a-gEGR1 vector
pU6
gCT
T5
pEF1
HA
Cas13a
mBN
MLS
Cas13a-gCT vector
B
Mitochondria-specific targeting
MLS
Cas13a
mBN
EGR1 ntmRNA
C
Mitochondrial isolation
RNA extraction & Quality control
RNA-seq
Differential RNAs
Cells
EGR1
MTND4P12
KIF26A
MT-RNR2
COL18A1
AC0071924
FBLN2
COL5A2
PCOLCE
MTND5P11
HL7702
HepG2
8
6
4
2
Cells
HepG2
HL7702

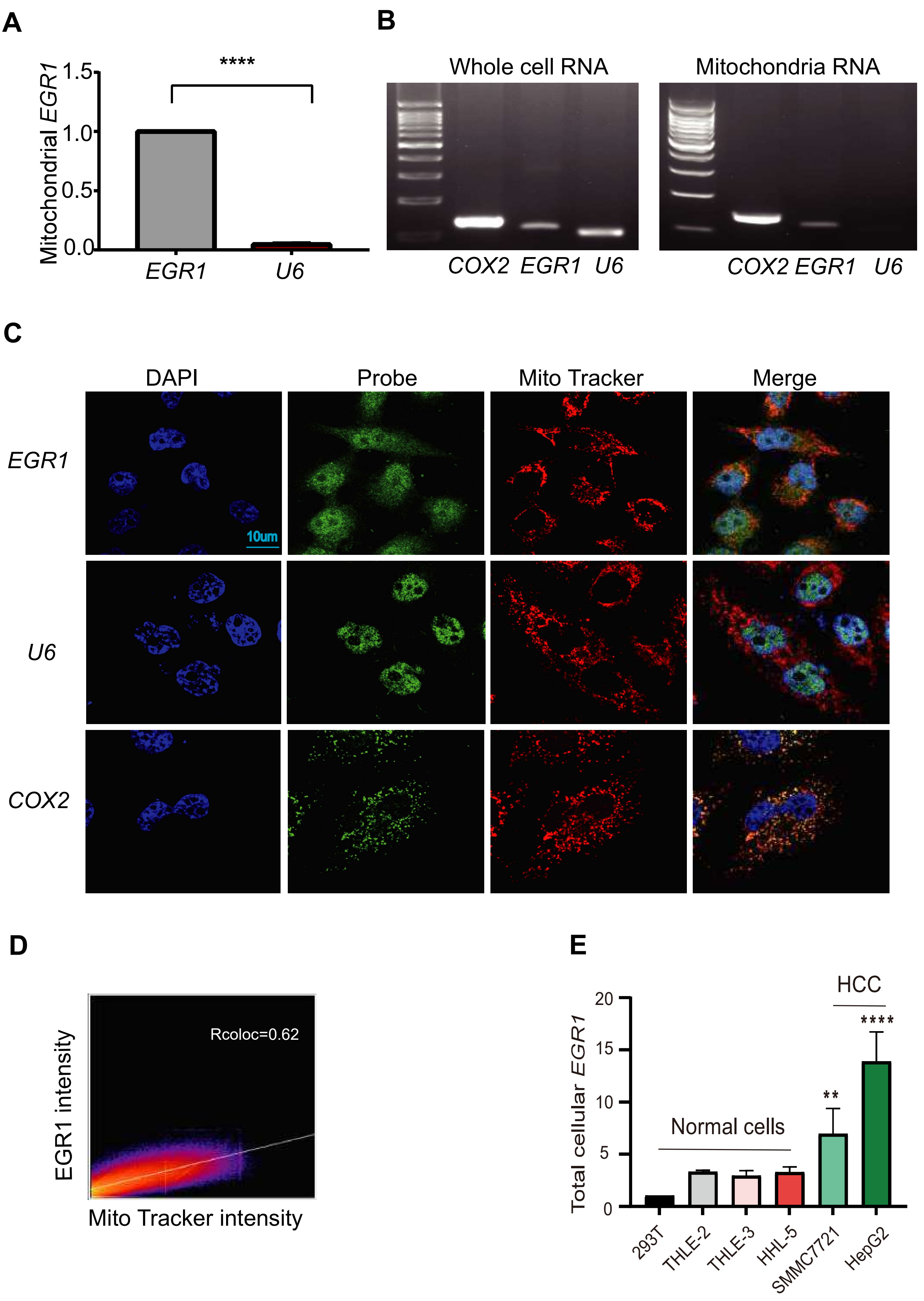
A
****
Mitochondrial EGR1
1.5
1.0
0.5
0.0
EGR1
U6
B
Whole cell RNA
Mitochondria RNA
COX2 EGR1 U6
COX2 EGR1 U6
C
DAPI
Probe
Mito Tracker
Merge
EGR1
10um
U6
COX2
D
Rcoloc=0.62
EGR1 intensity
Mito Tracker intensity
E
HCC
****
**
Normal cells
Total cellular EGR1
20
15
10
5
0
293T
THLE-2
THLE-3
HHL-5
SMMC7721
HepG2

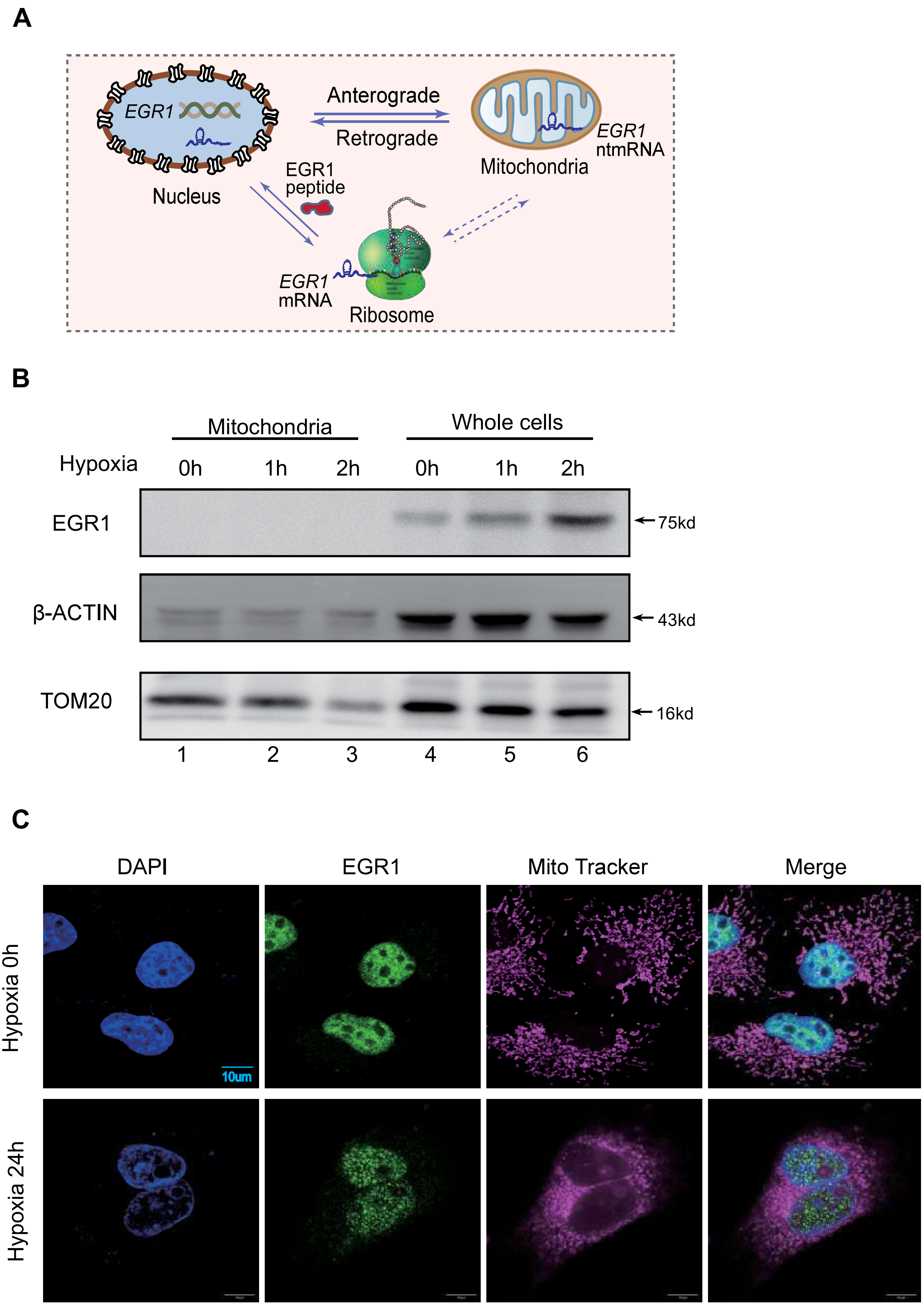
A
EGR1
Anterograde
Retrograde
EGR1
ntmRNA
Mitochondria
Nucleus
EGR1
peptide
EGR1
mRNA
Ribosome
B
Mitochondria
Whole cells
Hypoxia
0h
1h
2h
0h
1h
2h
EGR1
75kd
β-ACTIN
43kd
TOM20
16kd
1
2
3
4
5
6
C
DAPI
EGR1
Mito Tracker
Merge
Hypoxia 0h
10um
Hypoxia 24h

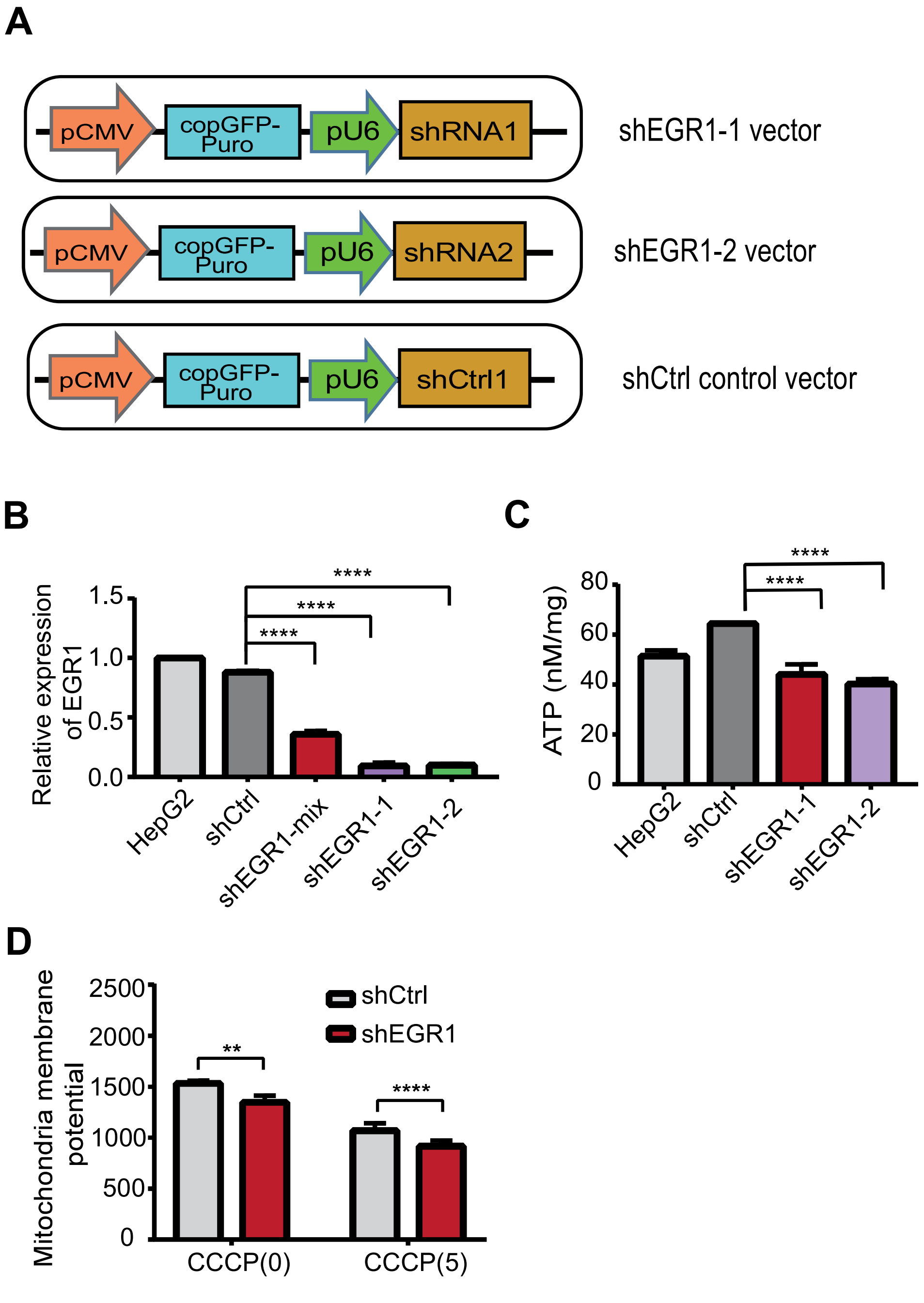
A
pCMV
copGFP-Puro
pU6
shRNA1
shEGR1-1 vector
pCMV
copGFP-Puro
pU6
shRNA2
shEGR1-2 vector
pCMV
copGFP-Puro
pU6
shCtrl1
shCtrl control vector
B
Relative expression of EGR1
1.5
1.0
0.5
0.0
****
****
****
HepG2
shCtrl
shEGR1-mix
shEGR1-1
shEGR1-2
C
ATP (nM/mg)
80
60
40
20
0
****
****
HepG2
shCtrl
shEGR1-1
shEGR1-2
D
Mitochondria membrane potential
2500
2000
1500
1000
500
0
shCtrl
shEGR1
**
****
CCCP(0)
CCCP(5)

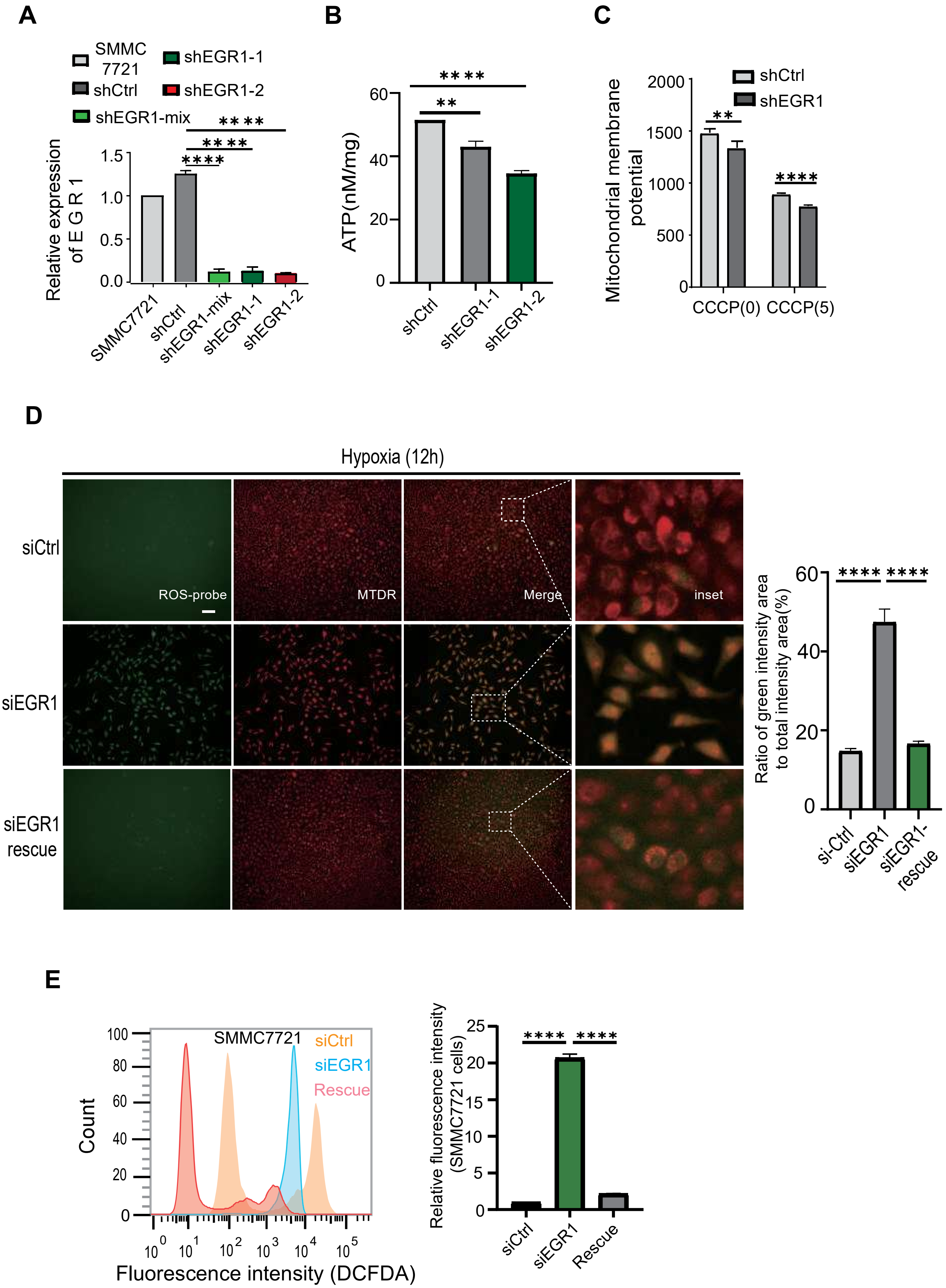
A
SMMC 7721
shCtrl
shEGR1-mix
shEGR1-1
shEGR1-2
Relative expression of E G R 1
SMMC7721
shCtrl
shEGR1-mix
shEGR1-1
shEGR1-2
B
ATP(nM/mg)
shCtrl
shEGR1-1
shEGR1-2
C
shCtrl
shEGR1
Mitochondrial membrane potential
CCCP(0)
CCCP(5)
D
Hypoxia (12h)
siCtrl
siEGR1
siEGR1 rescue
ROS-probe
MTDR
Merge
inset
Ratio of green intensity area to total intensity area(%)
si-Ctrl
siEGR1
siEGR1-rescue
E
SMMC7721
siCtrl
siEGR1
Rescue
Count
Fluorescence intensity (DCFDA)
Relative fluorescence intensity (SMMC7721 cells)
siCtrl
siEGR1
Rescue

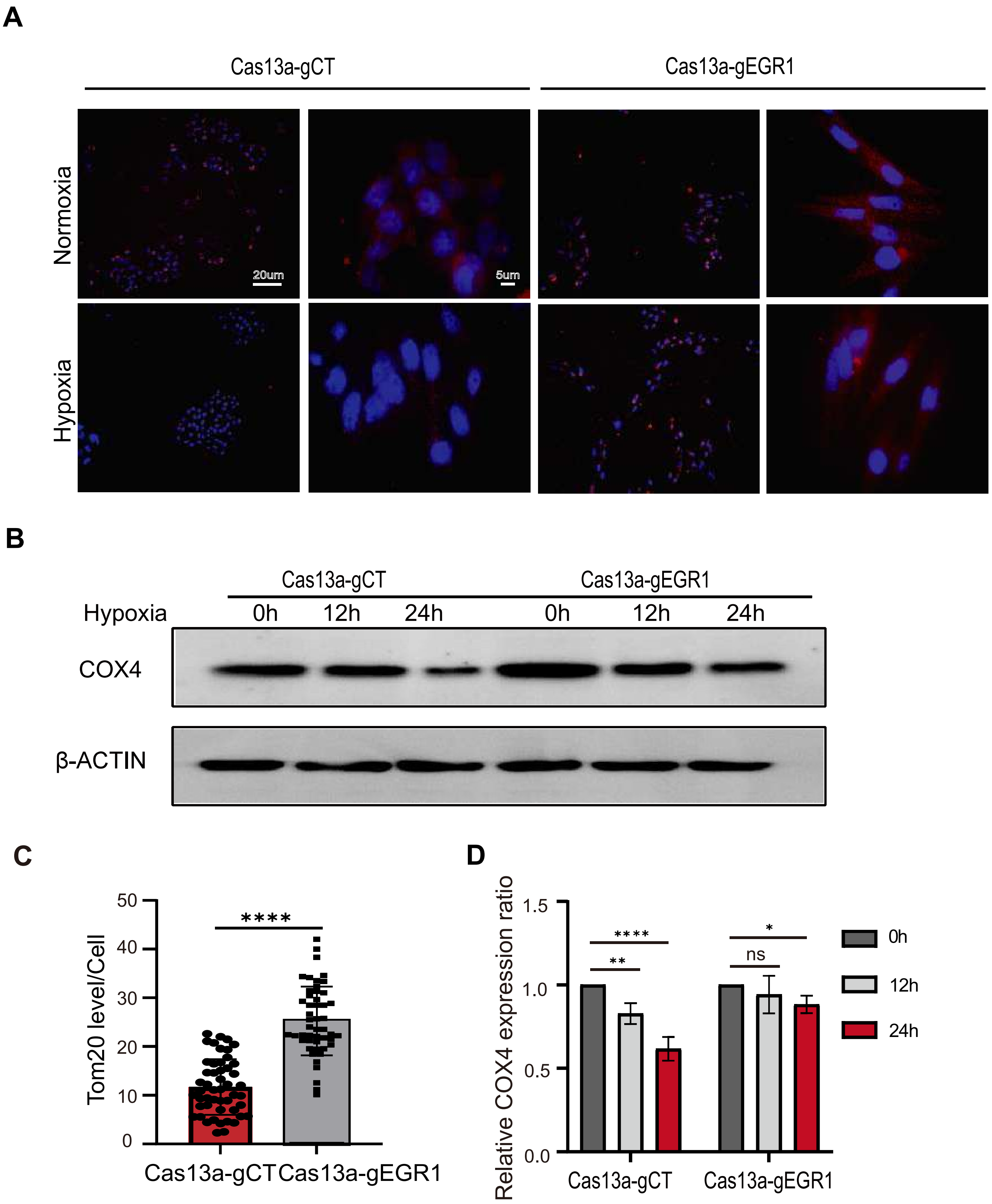

A
Cas13a-gCT
Cas13a-gEGR1
Normoxia
Hypoxia
20um
5um
B
Cas13a-gCT
Cas13a-gEGR1
Hypoxia
0h
12h
24h
0h
12h
24h
COX4
β-ACTIN
C
Tom20 level/Cell
****
Cas13a-gCT
Cas13a-gEGR1
D
Relative COX4 expression ratio
****
**
*
ns
0h
12h
24h
Cas13a-gCT
Cas13a-gEGR1

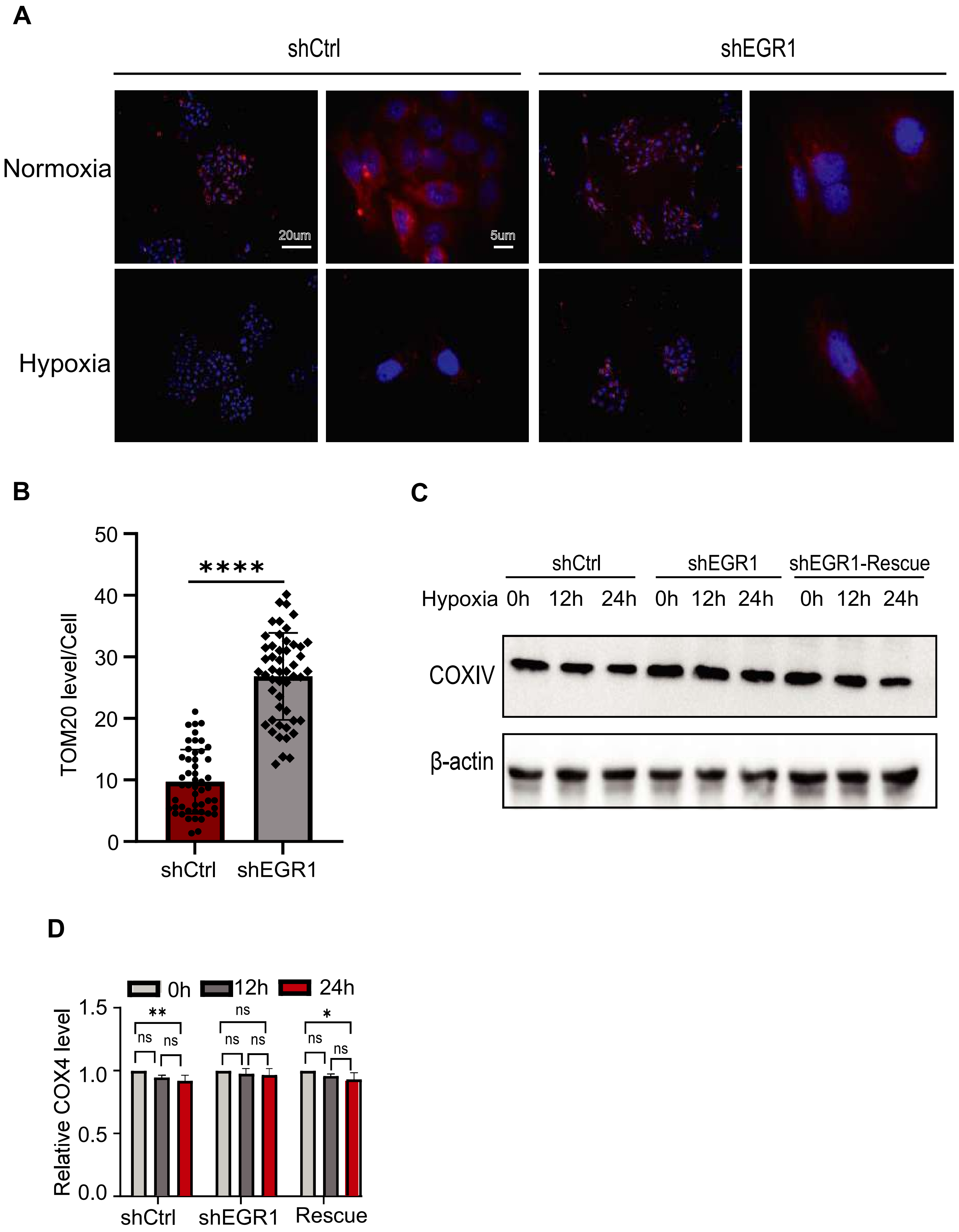
A
shCtrl
shEGR1
Normoxia
Hypoxia
20um
5um
B
TOM20 level/Cell
50
40
30
20
10
0
****
shCtrl
shEGR1
C
shCtrl
shEGR1
shEGR1-Rescue
Hypoxia 0h 12h 24h 0h 12h 24h 0h 12h 24h
COXIV
β-actin
D
0h
12h
24h
Relative COX4 level
1.5
1.0
0.5
0.0
**
ns
*
ns ns ns ns ns ns
shCtrl
shEGR1
Rescue

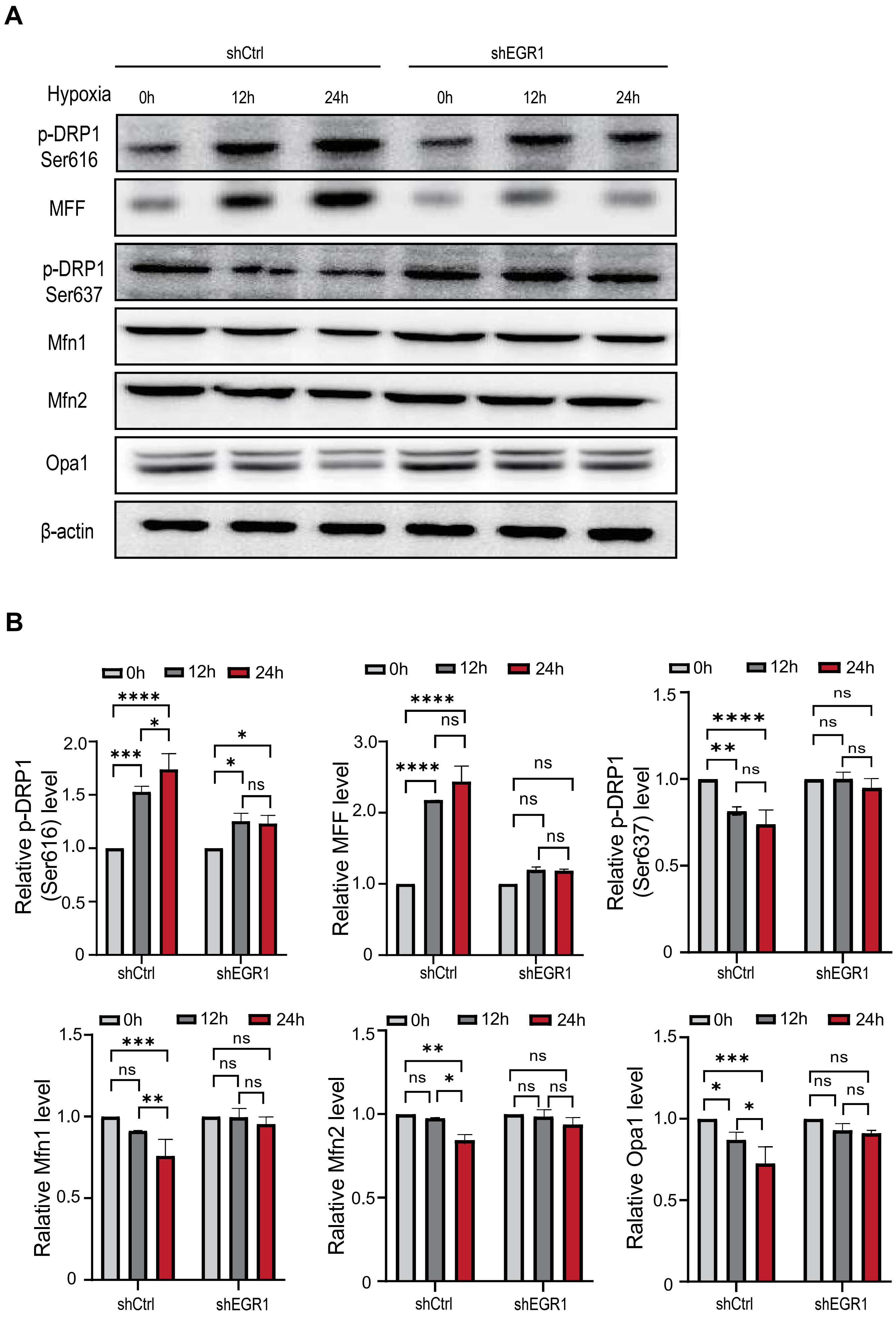
A
shCtrl
shEGR1
Hypoxia
0h
12h
24h
0h
12h
24h
p-DRP1 Ser616
MFF
p-DRP1 Ser637
Mfn1
Mfn2
Opa1
β-actin
B
0h
12h
24h
Relative p-DRP1 (Ser616) level
Relative MFF level
Relative p-DRP1 (Ser637) level
Ralative Mfn1 level
Ralative Mfn2 level
Ralative Opa1 level
shCtrl
shEGR1
ns

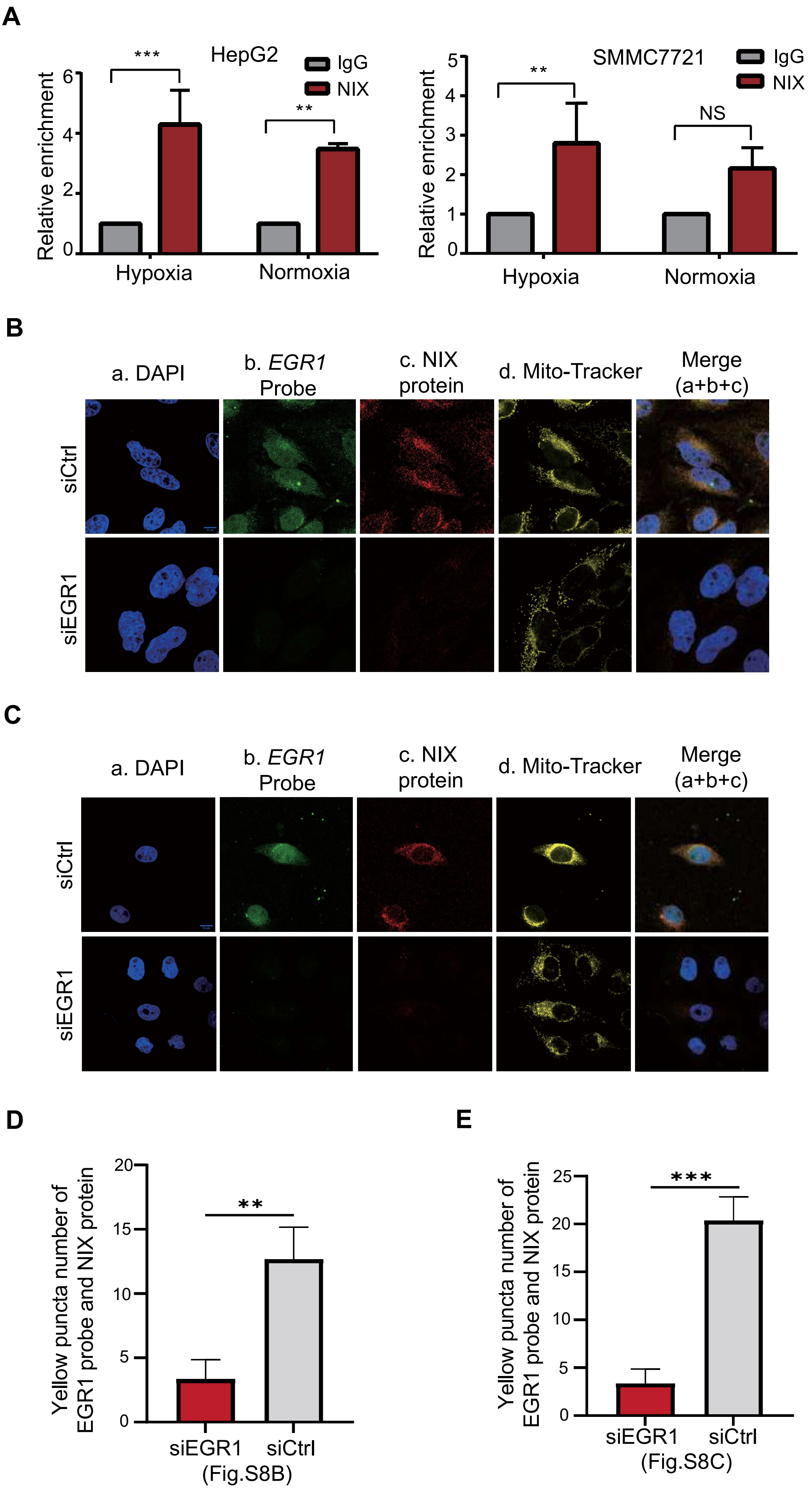
A
HepG2
IgG
NIX
***
**
Relative enrichment
0
2
4
6
Hypoxia
Normoxia
SMMC7721
IgG
NIX
**
NS
Relative enrichment
0
1
2
3
4
5
Hypoxia
Normoxia
B
a. DAPI
b. EGR1 Probe
c. NIX protein
d. Mito-Tracker
Merge (a+b+c)
siCtrl
siEGR1
C
a. DAPI
b. EGR1 Probe
c. NIX protein
d. Mito-Tracker
Merge (a+b+c)
siCtrl
siEGR1
D
Yellow puncta number of EGR1 probe and NIX protein
0
5
10
15
20
**
siEGR1
siCtrl
(Fig.S8B)
E
Yellow puncta number of EGR1 probe and NIX protein
0
5
10
15
20
25
***
siEGR1
siCtrl
(Fig.S8C)

A

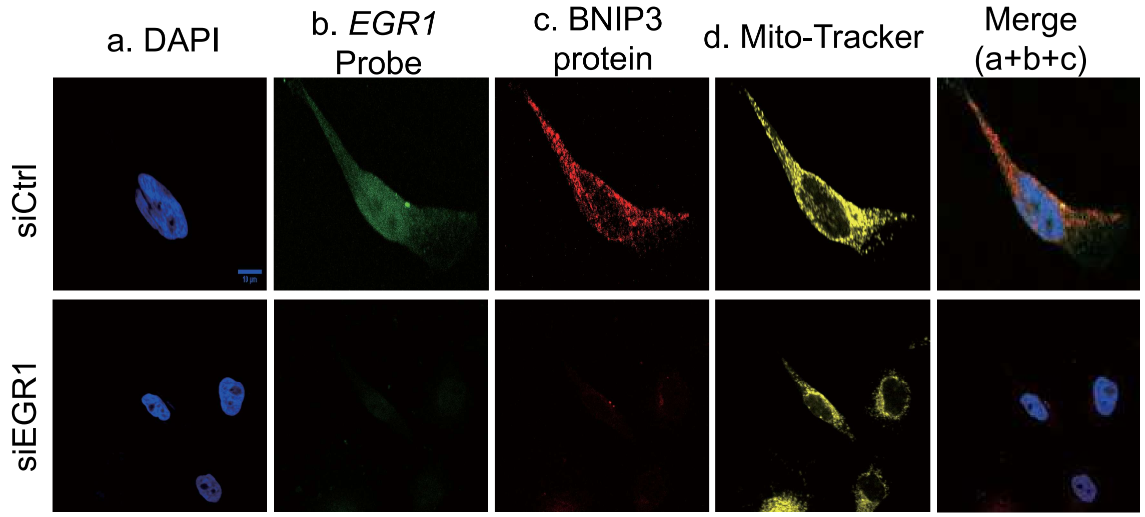
a. DAPI
b. EGR1 Probe
c. BNIP3 protein
d. Mito-Tracker
Merge (a+b+c)
siCtrl
siEGR1

B

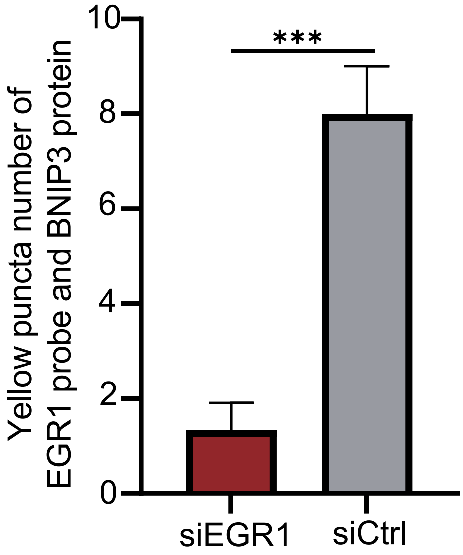
Yellow puncta number of EGR1 probe and BNIP3 protein
10
8
6
4
2
0
***
siEGR1
siCtrl

A

10	20	30	40	50	60
AAAAAGAAAA	GCCAAGCAAA	CCAATGGTGA	TCCTCTATTT	TGTGATGATG	CTGTGACAAT

70	80	90	100	110	120
AAGTTTGA**A**C	**C**TTT TTT TTT	GAAACAGCAG	TCCCAGTATT	CTCAGAGCAT	GTGTCAGAGT

130	140	150	160	169
GTTGTTCCGT	TAACCTT TTT	GTAAATACT**G**	CTTGACCGTA	CTCTCACAT

B

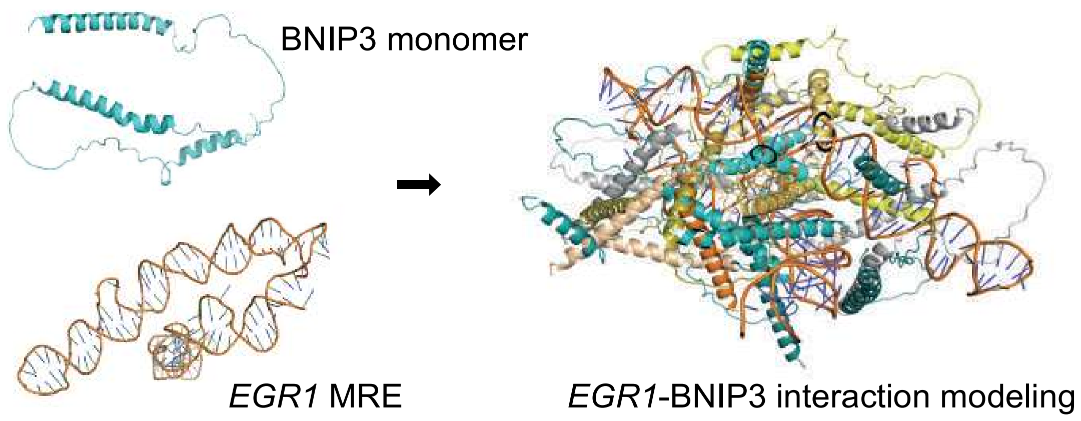

C

Models	Model-1	Model-2	Model-3	Model-4	Model-5	Model-6	Model-7	Model-8	Model-9	Model-10
Score	-372.36	-369.97	-362.06	-354.06	-351.48	-349.66	-332.1	-325.39	-323.06	-322.64
RMSD	159.6	178.48	176.43	169.82	155.53	158.78	168.11	164.72	135.64	159.13

D

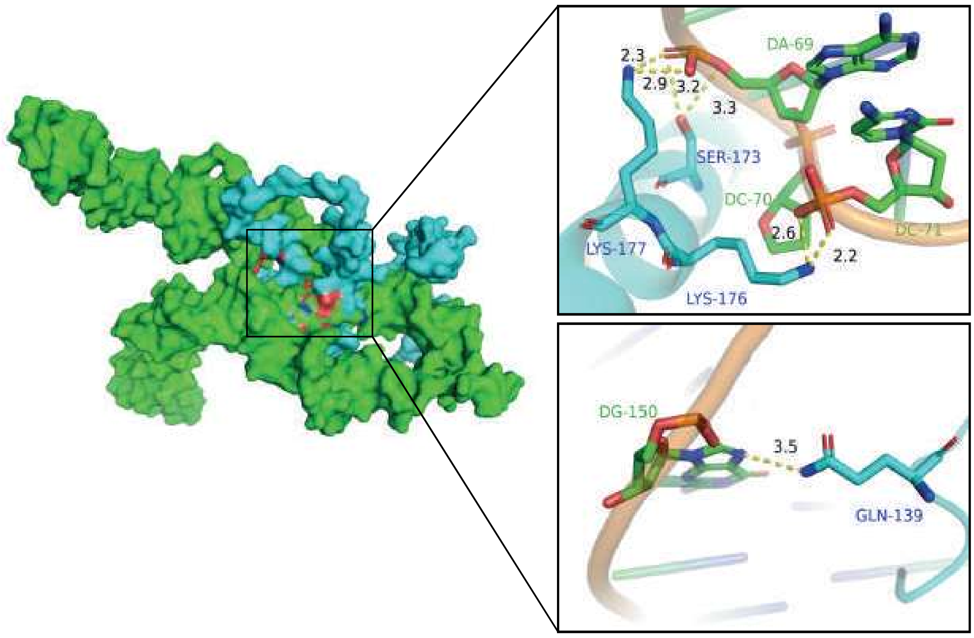

A

10	20	30	40	50	60
AAAAAGAAAA	GCCAAGCAAA	CCAATGGTGA	TCCTCTATTT	TGTGATGATG	CTGTG**A**CAA**T**
**70**	**80**	**90**	**100**	**110**	**120**
AAGTTTGAAC	CTTT TTT TTT	GAAACAGCAG	TCCCAGTATT	CTCAGAGC**AT**	GTGT**C**AGAGT
**130**	**140**	**150**	**160**	**169**	
GTTGTTCCGT	TAACCTT TTT	GTAAATACTG	CTT**GA**CCGTA	CTCTCACAT	

B

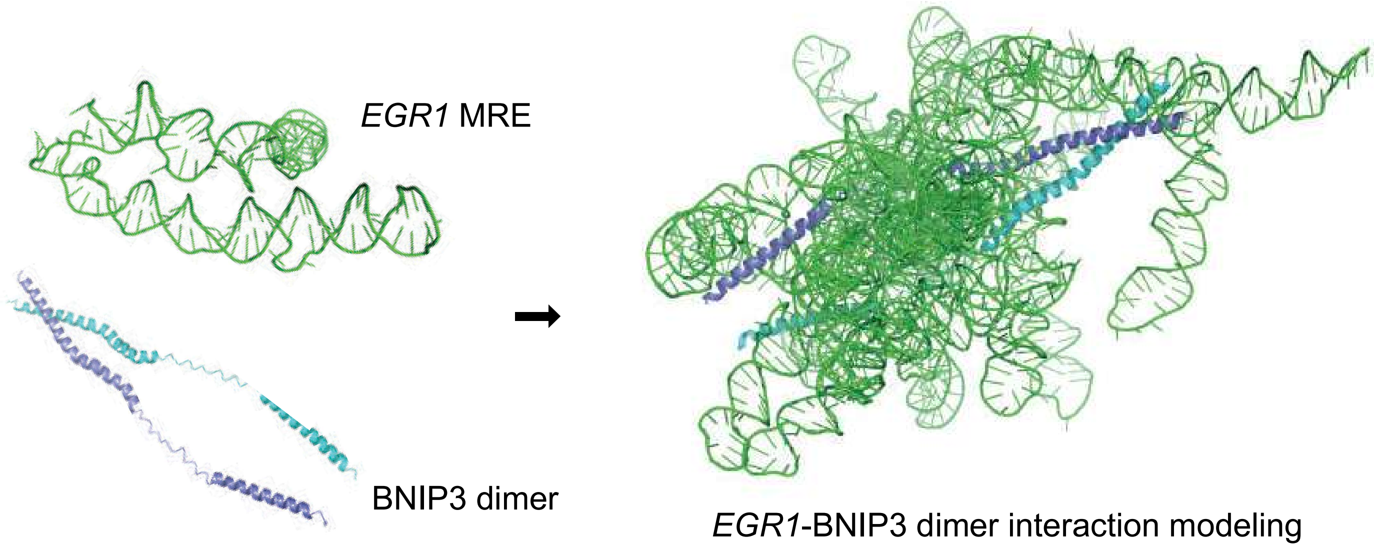

C

Models	Model-1	Model-2	Model-3	Model-4	Model-5	Model-6	Model-7	Model-8	Model-9	Model-10
Score	-423.95	-403.4	-402.16	-390.79	-385.84	-380.6	-376.24	-370.66	-367.68	-364.99
RMSD	180.08	196.26	165.55	169.26	171.77	186.5	167.92	165.82	184.55	163.48

A

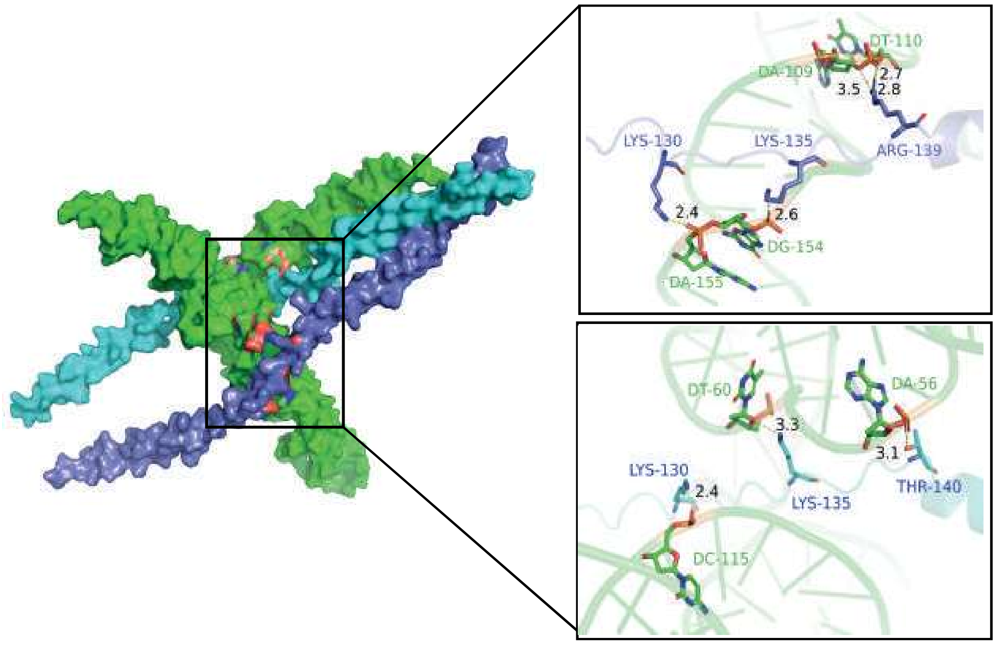

DT-110
DA-109
3.5
2.7
2.8
LYS-130
LYS-135
ARG-139
2.4
2.6
DG-154
DA-155
DT-60
DA-56
3.3
3.1
LYS-130
2.4
LYS-135
THR-140
DC-115

B

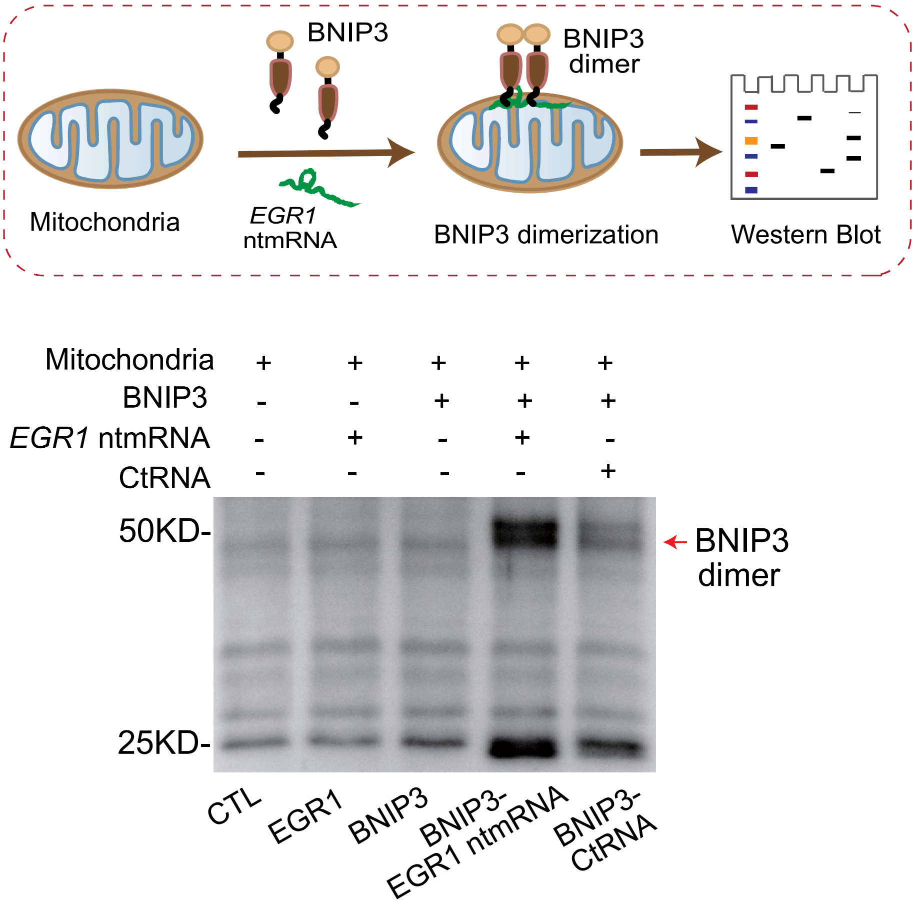

BNIP3
BNIP3 dimer
Mitochondria
EGR1 ntmRNA
BNIP3 dimerization
Western Blot
Mitochondria + + + + +
BNIP3 - - + + +
EGR1 ntmRNA - + - + -
CtRNA - - - - +
50KD-
25KD-
BNIP3 dimer
CTL
EGR1
BNIP3
BNIP3-EGR1 ntmRNA
BNIP3-CtRNA

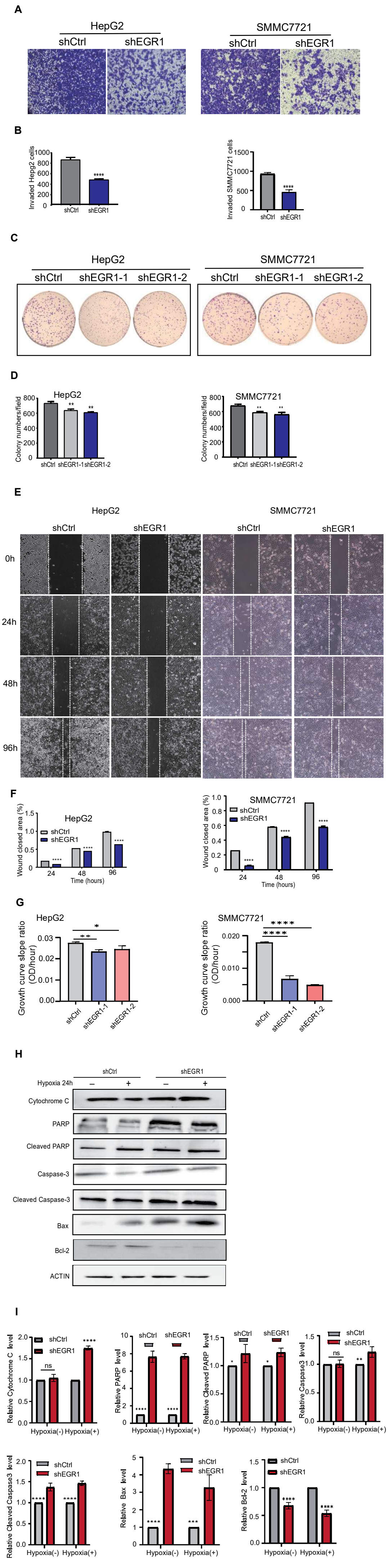
A
HepG2
shCtrl
shEGR1
SMMC7721
shCtrl
shEGR1
B
Invaded Hepg2 cells
shCtrl
shEGR1
****
Invaded SMMC7721 cells
shCtrl
shEGR1
****
C
HepG2
shCtrl
shEGR1-1
shEGR1-2
SMMC7721
shCtrl
shEGR1-1
shEGR1-2
D
HepG2
Colony numbers/field
shCtrl
shEGR1-1
shEGR1-2
SMMC7721
Colony numbers/field
E
HepG2
SMMC7721
shCtrl
shEGR1
0h
24h
48h
96h
F
HepG2
Wound closed area (%)
shCtrl
shEGR1
Time (hours)
SMMC7721
G
HepG2
Growth curve slope ratio (OD/hour)
shCtrl
shEGR1-1
shEGR1-2
SMMC7721
H
shCtrl
shEGR1
Hypoxia 24h
Cytochrome C
PARP
Cleaved PARP
Caspase-3
Cleaved Caspase-3
Bax
Bcl-2
ACTIN
I
Relative Cytochrome C level
Relative PARP level
Relative Cleaved PARP level
Relative Caspase3 level
Relative Cleaved Caspase3 level
Relative Bax level
Relative Bcl-2 level
shCtrl
shEGR1
ns
Hypoxia(-)
Hypoxia(+)

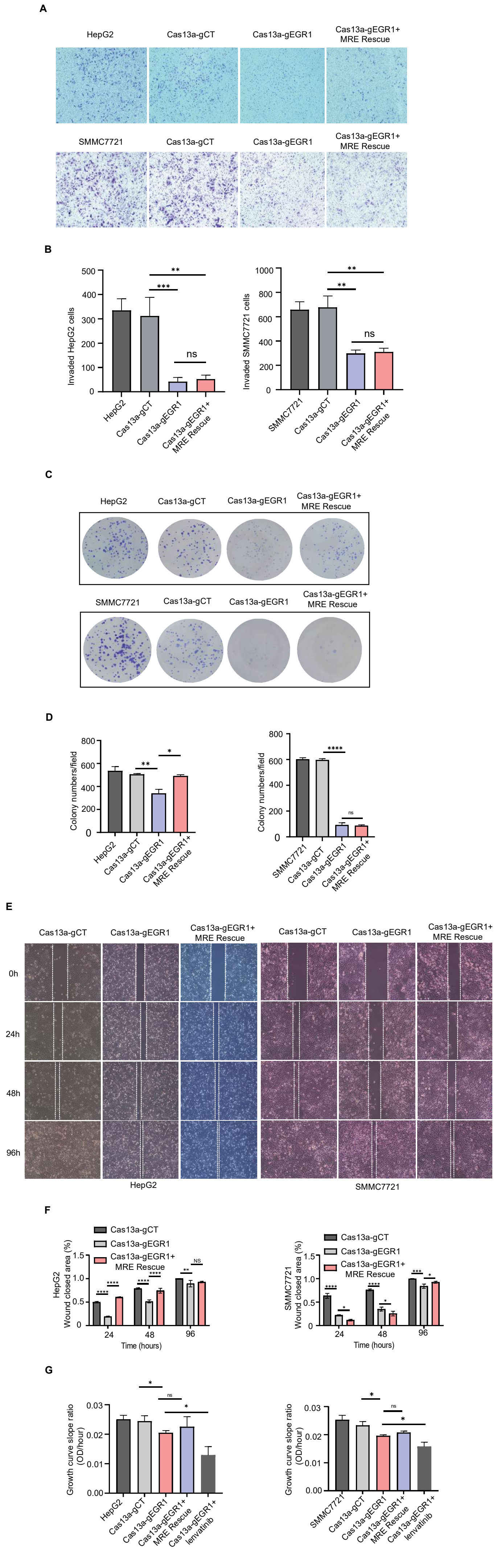
A
HepG2
Cas13a-gCT
Cas13a-gEGR1
Cas13a-gEGR1+
MRE Rescue
SMMC7721
B
Invaded HepG2 cells
Invaded SMMC7721 cells
ns
HepG2
SMMC7721
Cas13a-gCT
Cas13a-gEGR1
Cas13a-gEGR1+
MRE Rescue
C
D
Colony numbers/field
E
0h
24h
48h
96h
HepG2
SMMC7721
F
HepG2
Wound closed area (%)
SMMC7721
Time (hours)
G
Growth curve slope ratio
(OD/hour)
Cas13a-gEGR1+
lenvatinib
